# New methods for robust continuous wave T_1ρ_ relaxation preparation

**DOI:** 10.1002/nbm.4834

**Published:** 2022-10-07

**Authors:** Swetha Pala, Nina E. Hänninen, Olli Nykänen, Timo Liimatainen, Mikko J. Nissi

**Affiliations:** ^1^ Department of Applied Physics University of Eastern Finland Kuopio Finland; ^2^ Research Unit of Medical Imaging, Physics and Technology University of Oulu Oulu Finland; ^3^ Department of Radiology Oulu University Hospital Oulu Finland

**Keywords:** Bloch simulation, contrast, field inhomogeneity, rotating frame of reference, T_1ρ_ relaxation

## Abstract

Measurement of the longitudinal relaxation time in the rotating frame of reference (T_1ρ_) is sensitive to the fidelity of the main imaging magnetic field (B_0_) and that of the RF pulse (B_1_). The purpose of this study was to introduce methods for producing continuous wave (CW) T_1ρ_ contrast with improved robustness against field inhomogeneities and to compare the sensitivities of several existing and the novel T_1ρ_ contrast generation methods with the B_0_ and B_1_ field inhomogeneities. Four hard‐pulse and four adiabatic CW‐T_1ρ_ magnetization preparations were investigated. Bloch simulations and experimental measurements at different spin‐lock amplitudes under ideal and non‐ideal conditions, as well as theoretical analysis of the hard‐pulse preparations, were conducted to assess the sensitivity of the methods to field inhomogeneities, at low (ω_1_ << ΔB_0_) and high (ω_1_ >> ΔB_0_) spin‐locking field strengths. In simulations, previously reported single‐refocus and new triple‐refocus hard‐pulse and double‐refocus adiabatic preparation schemes were found to be the most robust. The mean normalized absolute deviation between the experimentally measured relaxation times under ideal and non‐ideal conditions was found to be smallest for the refocused preparation schemes and broadly in agreement with the sensitivities observed in simulations. Experimentally, all refocused preparations performed better than those that were non‐refocused. The findings promote the use of the previously reported hard‐pulse single‐refocus ΔB_0_ and B_1_ insensitive T_1ρ_ as a robust method with minimal RF energy deposition. The double‐refocus adiabatic B_1_ insensitive rotation‐4 CW‐T_1ρ_ preparation offers further improved insensitivity to field variations, but because of the extra RF deposition, may be preferred for ex vivo applications.

## Graphical abstract

New and existing state‐of‐the‐art CW‐T_1ρ_ schemes were compared theoretically, numerically, and experimentally at 9.4 T. Of the CW‐T_1ρ_ methods using hard pulses, the proposed triple‐focus and earlier single‐refocus methods were highly robust, while the scheme using adiabatic excitation and double refocusing with BIR‐4 was the most robust.

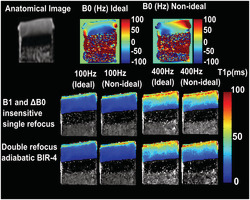

**Abbreviations used**
AFP: adiabatic full passage
AHP: adiabatic half passage
BIR: B_1_ insensitive rotation
B‐SL: balanced spin lock
CW‐T_1ρ_
: continuous wave T_1ρ_
FSE: fast spin echo
GRE: gradient echo
HS: hyperbolic secant
MNAD: mean normalized absolute deviation
PSC‐SL: paired self‐compensated spin lock
RF: radio frequency
RMS: root mean square
ROI: region of interest
SAR: specific absorption rate
SL: spin lock
SNR: signal‐to‐noise ratio
TE: echo time
WASSR: water saturation shift referencing
ω_1_
: external field (B1)
ω_eff_
: effective field

## 1 INTRODUCTION

Relaxation in the rotating frame under the presence of an external spin‐locking radio frequency (RF) pulse, termed T_1ρ_ relaxation,^1^ has been under active research for the quantitative assessment of different tissue types, such as the central nervous system,^2^ liver,^3^ and articular cartilage.^4^, ^5^ For instance, in articular cartilage, T_1ρ_ has been shown to be sensitive to the proteoglycan content, the collagen fiber network, and to degenerative changes in general.^5^, ^6^, ^7^, ^8^ T_1ρ_ relaxation depends on the amplitude of the spin‐lock (SL) pulse, that is, the SL frequency, which in typical cases corresponds to the timescales of slow molecular motion.^9^ In biological tissues, the processes affecting T_1ρ_ relaxation include dipolar interaction, chemical exchange, and the motion of spins through field gradients; broadly, any local fluctuations in the magnetic field that are on the same or lower frequency scale as the SL frequency.^8^, ^9^, ^10^, ^11^, ^12^ The relative importance of each mechanism varies with the SL frequency and the strength of the main magnetic field.^13^ The standard T_1ρ_ measurement uses on‐resonance continuous‐wave (CW) spin‐locking (CW‐T_1ρ_), and consists of tilting the magnetization 90 degrees and then locking the spins with a continuous RF pulse.^1^ Several methods to produce T_1ρ_ contrast at constant spin‐locking amplitude have been proposed, with variable sensitivity to the inhomogeneities of the main field (B_0_) and the RF field (B_1_).

Spin locking slows the relaxation process in the transverse plane by forcing the spins to rotate around the RF field. Because of the high sensitivity of the T_1ρ_ measurement to field inhomogeneities, the design of the SL pulse is essential for high quality T_1ρ_‐weighted images and accurate quantification of the T_1ρ_ relaxation time.^14^ Typically, in the clinical setting, the amplitudes of the SL pulses (ω_1_ = γB_1_/2π, where γ is the gyromagnetic ratio) are between a few hundred and a thousand Hz, most often 400–500 Hz. To allow estimation of the T_1ρ_ relaxation time, the same SL amplitude is maintained, while the SL durations are varied. The relaxation processes affecting T_1ρ_ are modulated by the molecular makeup of the tissue, and thus T_1ρ_ correlates with the properties of the tissues.^5^

Various methods have been reported for compensating the inherent sensitivity of T_1ρ_ measurement to field inhomogeneities.^14^, ^15^, ^16^ Witschey et al.^14^ introduced a T_1ρ_ weighting method, which was demonstrated to be highly insensitive to variations in the B_0_ and B_1_ fields, in phantoms and in vivo human brains at 3 T. The sequence is a modification of the ΔB_0_ insensitive SL sequence proposed by Zeng et al.,^17^ with a change to the phase of the final 90° pulse, effectively inverting the magnetization at the end of the preparation. While the pulse sequence was proven to be highly robust against B_0_ and B_1_ field inhomogeneities, the authors noted that the downside of the sequence was that it would still require a perfect 180° refocusing pulse to fully compensate against field variations. Another attempt to alleviate the sensitivity of spin locking to field inhomogeneities with a single‐refocus pulse, termed paired self‐compensated SL (PSC‐SL), was proposed by Mitrea et al.^15^ In their version, the spin‐locking periods were further split into pairs of opposite phases on either side of the refocusing pulse, making the SL pairs insensitive to B_1_ inhomogeneities; however, tiltin the magnetization back towards the positive z‐axis. The study demonstrated the sequence with phantom and small animal imaging at 7 T with gradient echo (GRE) and fast spin echo (FSE) readout sequences. A recent double‐refocusing pulse sequence, termed balanced SL (B‐SL), proposed by Gram et al.,^18^ applies an extra 180° refocusing pulse with opposite phase compensating for both inhomogeneities. The sequence was evaluated with simulations and demonstrated with an agarose phantom at 7 T. The authors concluded that B‐SL was superior in comparison with the existing single‐refocus sequence in which the magnetization is returned to the +Z axis, that is, the one presented by Zeng et al.^17^ However, it remains unclear how the B‐SL sequence performs in comparison with the sequence presented by Witschey et al.,^14^ which inverts the magnetization at the end of the preparation, as this sequence was also shown to be superior in comparison with the noninverting T_1ρ_ preparation.

Adiabatic pulses have also been used to improve the robustness of T_1ρ_ imaging. Various studies used adiabatic half passage (AHP) pulses, coupled to CW spin locking to improve the B_1_ robustness of the measurements^16^, ^19^, ^20^, ^21^, ^22^, ^23^ The AHP pulses were utilized in these studies for tilting the magnetization to the transverse plane for the CW SL, followed by a reverse AHP to bring the magnetization back to the longitudinal axis. A dual acquisition method was proposed by Chen^16^ to address the adverse effect from relaxation during the reverse AHP on T_1ρ_ quantification. The method was demonstrated with phantom and human liver imaging at 3 T. Similar methods, using pulsed, fully adiabatic T_1ρ_ preparation, have also been reported.^24^, ^25^, ^26^

The purpose of this study was twofold; firstly, to perform a numerical, experimental, and partial theoretical comparison of the sensitivities of the different T_1ρ_ contrast generation methods to the inhomogeneities in the B_1_ and B_0_ fields; and secondly, to introduce additional ways of producing T_1ρ_ contrast with reduced sensitivity to the field inhomogeneities. We examined the different previously published and new T_1ρ_ preparation methods via both Bloch simulations and experimentally. In the theoretical part, we focused on the different hard‐pulse implementations for T_1ρ_ preparation.

## 2 MATERIALS AND METHODS

### 2.1 CW‐T_1ρ_ preparation schemes

**FIGURE 1:**
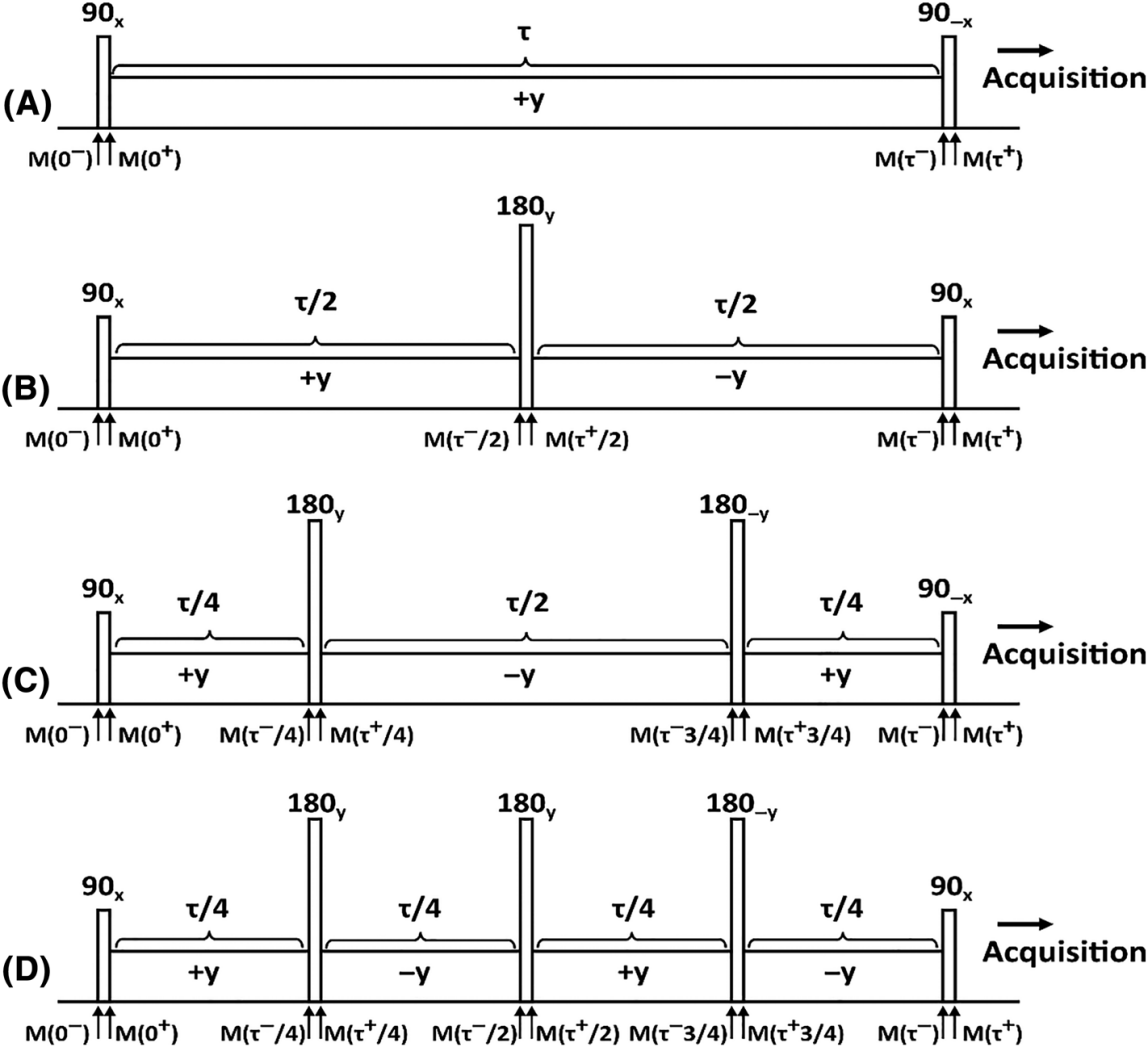
Hard‐pulse SL preparations. (A) Conventional SL,^14^ (B) Single‐refocus ΔB_0_ and B_1_ insensitive SL,^14^ (C) Double‐refocus “B‐SL”,^18^ and (D) Triple‐refocus CW‐T_1ρ_ schemes for T_1ρ_ contrast preparation. M(0^−^) is the initial magnetization before the excitation pulse. The magnetization is flipped from the longitudinal plane (z‐axis) towards the transverse plane by the first pulse; M(0^+^) is the magnetization after the excitation. During the SL (of duration τ), the magnetization nutates about the SL field, along the z''‐axis; after *n* refocusing pulses and spin‐locking segments, the magnetization is M(τ^−^) before the final rewinder pulse. The final magnetization (M[τ^+^]) is returned back to the longitudinal axis by the rewinder pulse. Please note that (A) and (C) return the magnetization to the +z axis with a reverse 90° pulse, while (B) and (D) take the magnetization to the ‐z axis with another forward 90° pulse. B‐SL, balanced spin lock; CW‐T_1ρ_, continuous wave T_1__ρ_; SL, spin lock

Here, we focus on the conventional non‐refocused hard‐pulse, single‐refocused ΔB_0_ and B_1_ insensitive preparation scheme presented by Witschey et al.,^14^ the double‐refocused B‐SL preparation scheme presented by Gram et al.,^18^ and on a novel triple‐refocused hard‐pulse CW‐T_1ρ_ preparation scheme. Triple refocused hard‐pulse CW‐T_1ρ_ attempts to account for the reported inability of the single‐refocus sequence presented by Witschey et al.,^14^ to fully compensate for the field variations if the single refocus is not a perfect 180° pulse (Figures 1, S1, and S2). Theoretical derivations on the sensitivities of the preparation are provided in the supporting information and in Witschey et al.^14^ In addition, the ΔB_0_ and B_1_ insensitive T_1ρ_ preparation presented by Mitrea et al.^15^ was considered in simulations.

**FIGURE 2:**
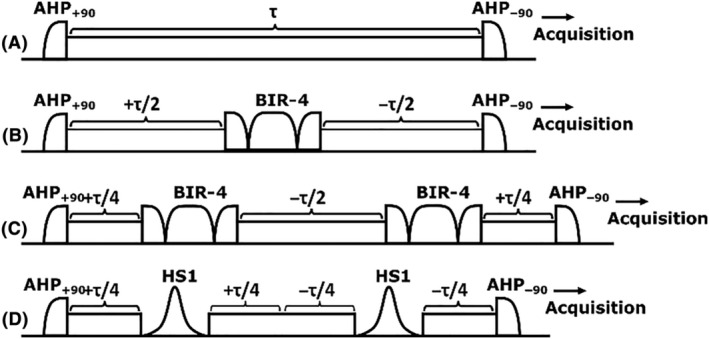
Adiabatic and CW SL preparations. (A) Conventional adiabatic CW‐T_1ρ_ preparation, consisting of an AHP excitation, a SL of duration τ, and a reverse AHP.^19^ Adiabatic CW‐T_1ρ_ with (B) A single adiabatic BIR‐4 refocusing pulse, (C) With two BIR‐4 refocusing pulses, or (D) Using double refocusing with HS1 pulses. The negative sign in front of τ indicates a phase shift of 180°. AHP, adiabatic full passage; BIR, B_1_ insensitive rotation; CW, continuous wave; CW‐T_1ρ_, continuous wave T_1ρ_; HS, hyperbolic secant; SL, spin lock

Adiabatic pulses are amplitude‐ and frequency‐modulated RF pulses that are highly insensitive to B_1_ inhomogeneity and off‐resonance effects.^27^ In adiabatic pulses, the amplitude of the effective field (ω_eff_ [t]) of the pulse is the vectorial sum of the time‐dependent B_1_ and the off‐resonance component. The flip angle (φ) is largely independent of the applied B_1_ field, given that the adiabatic condition |ω_eff_ (t)|>> |dφ / dt| is satisfied, that is, the sweep of the direction of the effective field (dφ/dt) is slow compared with its amplitude (ω_eff_). During an adiabatic sweep, spins at different resonances are primarily affected at different times of the pulse, in contrast to the CW‐pulse, which simultaneously affects the spins within its frequency bandwidth. Adiabatic pulses can be categorized as excitation, refocusing, and inversion pulses.^28^ AHP pulses (Figure 2A) are employed to generate uniform excitation with a 90° flip on a defined frequency band, leaving the magnetization in the transverse plane, while reverse AHP pulses brings the magnetization back to the *z* axis from the transverse plane.^19^ With the adiabatic excitation and CW‐SL, the SL continues from the same phase where the adiabatic excitation pulse ends, but the amplitude of the RF pulse is reduced to the desired spin‐lock amplitude (i.e., unlike in the adiabatic CW T_1ρ_ reported by Chen,^16^ where the amplitude of the SL equals the maximum amplitude of the AHP). Similarly, the reverse AHP starts from the phase where the SL ends, with amplitude ramped up to the maximum of the AHP.^16^, ^19^, ^22^, ^24^

Besides AHP excitation pulses, either B_1_ insensitive rotation (BIR)‐4 plane rotation pulses or adiabatic full passage (AFP) inversion pulses, such as hyperbolic secant (HS)*n* pulses, can be used for adiabatic refocusing/inversion during the spin‐locking train, both providing largely B_1_‐insensitive means for the refocusing/inversion.^28^, ^29^ As long as the adiabaticity can be sufficiently maintained during the pulses, inhomogeneities in the B_1_ field will not have an effect on the resulting flip angles using the adiabatic pulses. Here, we investigated four different CW‐T_1ρ_ preparations utilizing AFP, AHP, BIR‐4, and HS1 adiabatic pulses, without refocusing^22^ or using single or double BIR‐4 refocusing, or double AFP inversion, in between the SL (Figure 2).

### 2.2 Numerical simulations

Numerical Bloch simulations of the pulse trains were performed for ΔB_0_ and B_1_ field inhomogeneities of up to ±1 kHz and ±40%, respectively, to analyze the sensitivities of the sequences. The simulations for all the spin locking schemes were performed using SL durations of 8, 32, and 128 ms and SL amplitudes of 100 and 400 Hz. The duration of each of the hard 90° and 180° pulses was 200 μs. Maximum amplitudes of the adiabatic pulses were set to 2.5 kHz and the durations were 4, 3.03, and 5.17 ms for AHP, AFP, and BIR‐4, respectively. Additionally, conventional adiabatic CW T_1ρ_ was simulated with a longer and lower maximum RF amplitude of 600‐Hz of the AHP pulses.^16^ The following modulation functions were used for adiabatic pulses: the AHP and BIR‐4 pulses utilized tanh/tan modulations^30^ and the AFP pulse was an HS1 pulse with a time‐bandwidth product value (R = 20). Relaxation effects were neglected in the simulations to focus on the effects of field inhomogeneity.

### 2.3 Sample preparation

Cylindrical osteochondral plugs (n = 4, diameter = 6 mm) were prepared from the patella of bovine knee joints obtained from a local grocery store. The samples were immersed in phosphate buffered saline containing enzyme inhibitors and frozen at −20°C. Prior to imaging, the samples were thawed and transferred into a custom‐built sample holder and test tube filled with perfluoropolyether (Galden HS‐240, Solvay Solexis, Italy). In addition to osteochondral plugs, cherry tomatoes (n = 2) and an agarose phantom (n = 1) were used as test samples. The cherry tomatoes were chosen such that they neatly fit within the RF coil. The cherry tomatoes were placed into the coil without immersion solution. The agarose phantom was prepared with 3% w/v agarose and water by heating the solution at 90°C. The agar solution was then transferred to a test tube and placed into a refrigerator (at ~ 5°C) for cooling and gel formation. The test tube was taken out of the refrigerator then allowed to settle to room temperature for 2 h prior to imaging.

### 2.4 MR imaging

MRI studies were performed using a 9.4‐T preclinical Varian/Agilent scanner (Vnmrj DirectDrive console v. 3.1) and a 19‐mm quadrature RF volume transceiver (Rapid Biomedical GmbH, Rimpar, Germany). A set of RF shapes for all the methods shown in Figures 1 and 2 for generating T_1ρ_ contrast was created for the experiments. All the CW‐T_1ρ_ measurements were conducted using a magnetization preparation block consisting of the RF train and a crusher gradient coupled to an FSE readout sequence. For each of the CW‐T_1ρ_ methods, five SL amplitudes (γB_1_/2π = 0, 50, 100, 200, and 400 Hz) were used. Hard 90° and 180° pulses were both set to have a duration of 200 μs and the adiabatic refocusing/inversion pulses used were BIR‐4 and HS1, with durations of 5.17 and 3.03 ms, respectively. The AHP pulse duration was 4 ms. All the adiabatic pulses (Figure 2) were set to have a maximum B_1_ amplitude of 2.5 kHz. All the T_1ρ_ measurements were performed using SL (CW) durations of 0, 4, 8, 16, 32, 64, 128, and 192 ms. In addition to T_1ρ_ measurements, a B_0_ map was acquired using the same FSE readout sequence, coupled to a water saturation shift referencing (WASSR)^31^ preparation module utilizing a saturation range of −300 to +300 Hz with a 50‐Hz step and saturation power of 30 Hz. Furthermore, the B_1_ field was estimated using a set of hard‐pulse saturation preparations around the expected 90° power (±40% from the expected power), coupled to a low‐resolution scan with the same FSE readout. The scan time for each of the aforementioned T_1ρ_ setups was ~ 48 min, for WASSR ~ 8 min, and for the B_1_ scan ~ 13 min. The parameters of the readout FSE sequence varied slightly depending on the sample and its size (Table 1).

**TABLE 1:** Measurement parameters besides spin locking for the experimental samples

Samples	Slice	TR	Resolution	FOV	B_0_ field inhomogeneity simulation	B_1_ field inhomogeneity simulation
Bone cartilage specimens	1 mm	5 s	256 x 128	16 x 16 mm	a	b
Tomato 1	4 mm	5 s	256 x 128	25 x 25 mm	a	c
Tomato 2	1 mm	5 s	256 x 128	20 x 30 mm	a	d
Agarose phantom	2 mm	5 s	256 x 128	40 x 15 mm	a	d

**a.** As‐good‐as‐possible shimming and then setting the shim gradients deliberately to incorrect values along a specific axis (phase‐encoding direction).

**b.** B_1_ amplitude increased by 20% from the calibrated amplitude.

**c.** The sample was moved about 15 mm away from the RF center (uniform field about 25 mm) to introduce an inhomogeneous B_1_ field.

**d.** The sample was larger than the homogenous RF region.

The samples were scanned under two nominal conditions: (i) as homogenous B_0_ and B_1_ as possible; and (ii) altered B_0_ and B_1_ settings to introduce inhomogeneities. At the beginning of every session, manual shimming of B_0_ and a calibration of the B_1_ transmit power was performed.

The measurements were first conducted for case (i) with as good conditions and homogenous fields as possible, and subsequently for case (ii) with the shims deliberately set to an incorrect value along a specific axis to induce B_0_ variation of approximately ±250 Hz along the chosen direction (in‐plane, across the cartilage surface for osteochondral samples, and along the same axis for the other samples). Additionally, the B_1_ amplitude was either set to 20% lower or higher than the nominal calibrated value, or the specimen was pulled approximately 15 mm away from the RF center (approximately 50% of the RF visibility range) so that the B_1_ field along the sample became inhomogeneous. For those specimens that exceeded the homogenous region of the B_1_ field, no additional B_1_ inhomogeneities were introduced (Table 1).

### 2.5 Data analysis

The results of the simulations were evaluated visually and semiquantitatively. For ΔB_0_ response with a correct B_1_ value and for ΔB_1_ response with correct B_0_, a semiquantitative metric was estimated: the width of the flat region of the response, that is, the width of the relatively smooth and flat response around the on‐resonance condition after applying a moving average window of 50 Hz width and a threshold of 90% of the on‐resonance amplitude. The averaging window width was changed to 10 Hz for the nonrefocused schemes and simulations of ΔB_0_ response at 100‐Hz SL amplitude to obtain reliable estimates. The results were calculated and visualized using the absolute values of the simulated *z* magnetization to facilitate comparison between the preparation schemes, because some of them deliberately take the magnetization to the ‐*z* axis.

Relaxation time maps were fitted in a pixel‐wise manner using the three‐parameter monoexponential fit, using in‐house developed plugins for Aedes (http://aedes.uef.fi) in Matlab (Matlab R2019b; MathWorks, Natick, MA, USA). B_0_ maps were calculated using Lorenzian fits to the acquired WASSR saturation datasets^31^ and the B_1_ maps were estimated via linear fitting to the acquired saturation datasets.

To compare the reliability and robustness of the different T_1ρ_ preparation schemes, mean normalized absolute deviation (MNAD) values in large regions of interest (ROIs) were calculated for each of the preparation schemes between the relaxation times measured under ideal and non‐ideal conditions. The large ROIs for each specimen were defined on an average T_1ρ_ map calculated over all the preparation schemes for the SL amplitude of 400 Hz. These ROIs, comprising areas with high SNR, were then used to extract the T_1ρ_ values from all the measurements under both conditions for further computations. The MNADs of the relaxation times were calculated by

(1) $$\text{MNAD}\;\left(T1\rho_{\text{nonideal}},T1\rho_{\text{ideal}}\;\right)=\text{mean}\left(\frac{|T1\rho_{\text{nonideal}}\left(i\right)-T1\rho_{\text{ideal}}\left(i\right)|}{\left(T1\rho_{\text{nonideal}}\left(i\right)+T1\rho_{\text{ideal}}\left(i\right)\right)/2}\right),$$

where *i* refers to an individual voxel within the ROIs under ideal and non‐ideal conditions. The MNAD value of 0.5 corresponds to a mean deviation of 50% of the T_1ρ_ relaxation times under the nonideal conditions. For the comparison of the different T_1ρ_ preparation schemes, MNAD values from all the samples available for a given preparation were averaged.

In addition to the primary spin‐locking pulse, each of the T_1ρ_ preparation schemes requires other RF pulses to tilt and refocus the magnetization. Depending on the configuration, the RF power deposited by these additional pulses varies significantly. To assess the relative differences in RF energy deposition between the preparations, root mean square (RMS) integrals of the pulse trains with zero SL duration were calculated. To facilitate the comparison, the RMS values were normalized with that of the conventional CW‐T_1ρ_ preparation.

## 3 RESULTS

**FIGURE 3:**
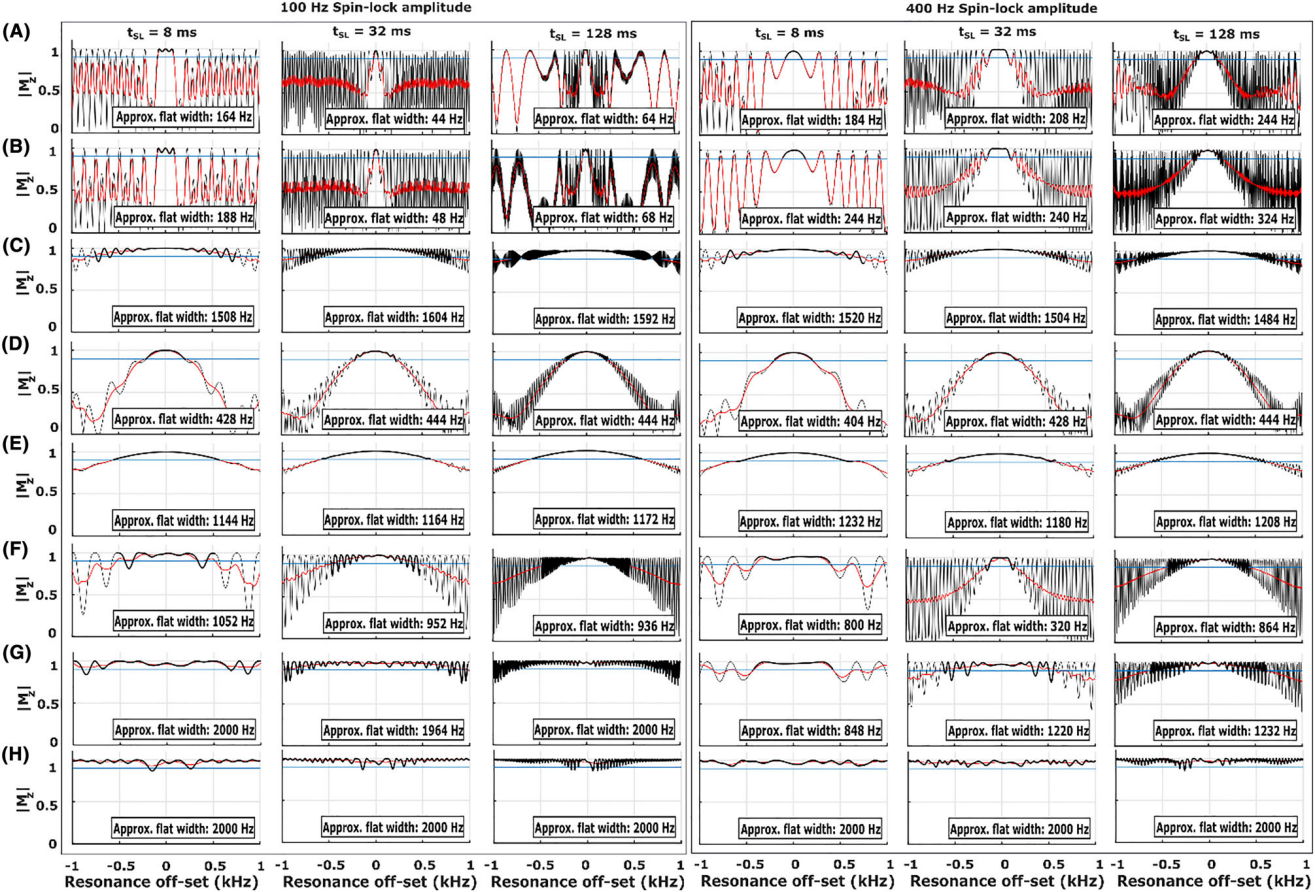
Simulations of the pulse sequences in Figures 1 and 2 with SL durations τ = 8, 32, and 128 ms and SL amplitudes of 100 and 400 Hz. The plots show Bloch simulations over ΔB_0_ (±1 kHz) at the correct B_1_ amplitude together with an estimated approximate flat response region (please see the Materials and Methods section) along the off‐resonance axis. (A) Conventional spin lock, (B) Conventional adiabatic CW‐T_1ρ_ spin lock, (C) Single‐refocus ΔB_0_ and B_1_ insensitive spin lock, (D) Double‐refocus B‐SL, (E) Triple‐refocus CW‐T_1ρ_, (F) Adiabatic CW‐T_1ρ_ with a single BIR‐4 refocusing, (G) Adiabatic CW‐T_1ρ_ with double BIR‐4 refocusing, and (H) Adiabatic CW‐T_1ρ_ with double HS1 refocusing. Absolute values of M_z_ are used to facilitate comparison between the sequences. B‐SL, balanced spin lock; BIR, B_1_ insensitive rotation; CW‐T_1ρ_, continuous wave T_1ρ_; HS, hyperbolic secant; SL, spin lock

**FIGURE 4:**
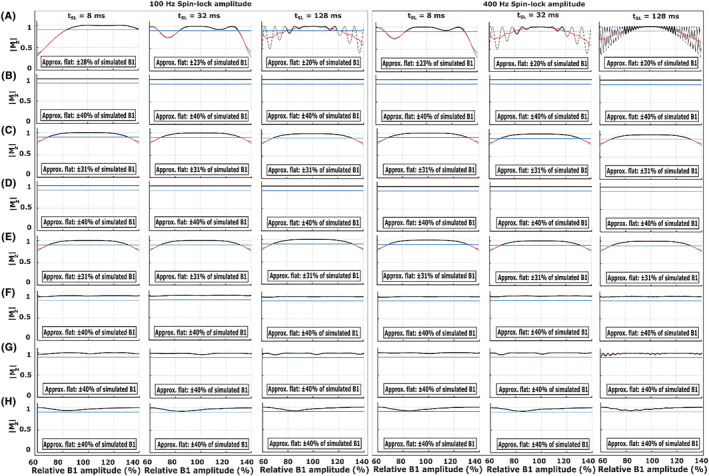
Simulations of the pulse sequences in Figures 1 and 2 with SL durations τ = 8, 32, and 128 ms and spin‐lock amplitudes of 100 and 400 Hz. The plots show Bloch simulations over ΔB_1_ (±40%) at the correct B_0_, together with an estimated approximate flat response region (please see the Materials and Methods section) along the relative B_1_ amplitude axis. (A) Conventional spin lock, (B) Conventional adiabatic CW‐T_1ρ_ spin lock, (C) Single‐refocus ΔB_0_ and B_1_ insensitive spin lock, (D) Double‐refocus B‐SL, (E) Triple‐refocus CW‐T_1ρ_, (F) Adiabatic CW‐T_1ρ_ with a single BIR‐4 refocusing, (G) Adiabatic CW‐T_1ρ_ with double BIR‐4 refocusing, and (H) Adiabatic CW‐T_1ρ_ with double HS1 refocusing. Absolute values of M_z_ are used to facilitate comparison between the sequences. B‐SL, balanced spin lock; BIR, B_1_ insensitive rotation; CW‐T_1ρ_, continuous wave T_1ρ_; HS, hyperbolic secant; SL, spin lock

Numerical simulations demonstrated variable sensitivity of the sequences to a range of offsets in the B_0_ and B_1_ fields (Figures 3, 4, and S3). 2D plots of the simulated responses on both ΔB_0_ and B_1_ offset axes demonstrate the differences in the sensitivities of the T_1ρ_ preparations: adiabatic refocused schemes demonstrated the least B_1_‐dependent variation and especially the double‐refocused versions also minimal ΔB_0_‐dependent variation at all simulated SL amplitudes (100 and 400 Hz) and SL times (8, 32, and 128 ms) (Figures 3F‐H and 4F‐H).

Quantification of the flatness of the simulated ΔB_0_ and ΔB_1_ responses at the nominally correct B_1_ and B_0_ indicated that the non‐refocused schemes had a very poor B_0_ off‐resonance response with almost no flat region even at the correct B_1_, while the refocused versions showed significantly improved responses (Figures 3C‐H, 4C‐H, S7 and S8). However, the adiabatic CW pulse simulated at 600‐Hz maximum amplitude (Figure S3B) had a broader flat response for both B_0_ and B_1_ inhomogeneities at the higher SL amplitude (400 Hz) (Figures S3B and S9) when compared with the 2.5‐kHz maximum amplitude simulations of the pulse (Figures 3, 4, and S7‐S9).

The adiabatic double‐refocused schemes had the broadest ΔB_0_ robustness, with the flat range essentially covering the entire simulated range from −1 to +1 kHz (and beyond), while the single‐ and triple‐refocused preparations had the broadest flat responses among the hard‐pulse preparation schemes (Figures 3C,E and 4C,E), but with a slight drop at B1 amplitudes beyond ±31% of the nominally correct amplitude. The double‐refocused hard pulse was highly insensitive to a wide range of B_1_ offsets, but was more sensitive to B_0_ inhomogeneities, being the least robust among the refocused schemes (Figures 3D, 4D, S7 and S8).

**FIGURE 5:**
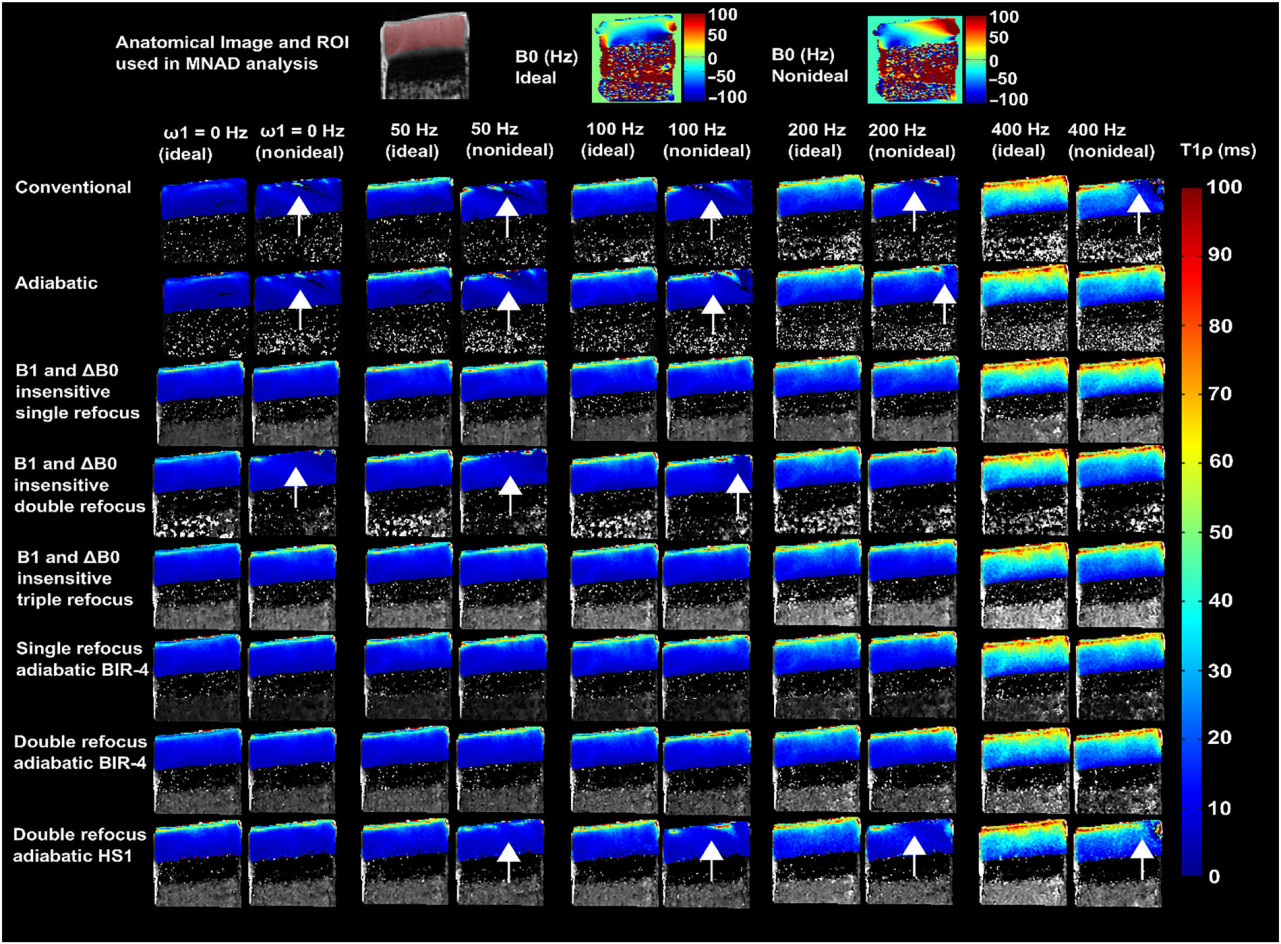
Representative examples of T_1ρ_ relaxation time maps of cartilage bone specimens under ideal and non‐ideal conditions with inhomogeneous B_0_ and B_1_ fields, for SL amplitudes of 0–400 Hz acquired with the different methods. Anatomical reference (showing the MNAD analysis ROI with red shading) and the corresponding B_0_ maps are shown at the top. Under the ideal conditions, all the methods provided largely artifact‐free T_1ρ_ relaxation time maps at all SL amplitudes. Under the non‐ideal conditions, the non‐refocused T_1ρ_ methods in particular performed poorly at lower SL amplitudes, while the refocused methods provided mostly artifact‐free relaxation time maps at all SL amplitudes. In particular, the double‐refocused adiabatic BIR‐4 method was robust. The arrows indicate locations where differences (artifacts) can be noted between the conditions. BIR, B1 insensitive rotation; MNAD, mean normalized absolute deviation; ROI, region of interest; SL, spin lock

**FIGURE 6:**
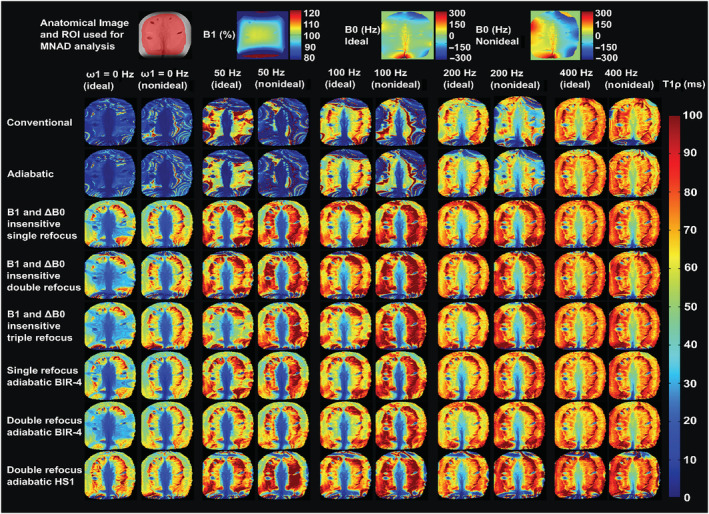
T_1ρ_ relaxation time maps of a cherry tomato sample, under as ideal as possible conditions and under non‐ideal conditions, with inhomogeneous B_0_ field, for SL amplitudes of 0–400 Hz acquired with the different methods. Anatomical reference (showing the MNAD analysis ROI with red shading) and the corresponding B_1_ and B_0_ maps are shown at the top. Under the ideal conditions, all the refocused methods provided largely artifact‐free T_1ρ_ relaxation time maps at all SL amplitudes, while the nonrefocused methods showed artifacts at the edges of the FOV at low SL amplitudes. Under the non‐ideal conditions, the nonrefocused T_1ρ_ methods in particular performed poorly at lower SL amplitudes, while the refocused methods provided mostly artifact‐free relaxation time maps at all SL amplitudes. The differences between the ideal and nonideal conditions can particularly be seen at the top and bottom edges with more significant field inhomogeneities. FOV, field of view; MNAD, mean normalized absolute deviation; ROI, region of interest; SL, spin lock

**FIGURE 7:**
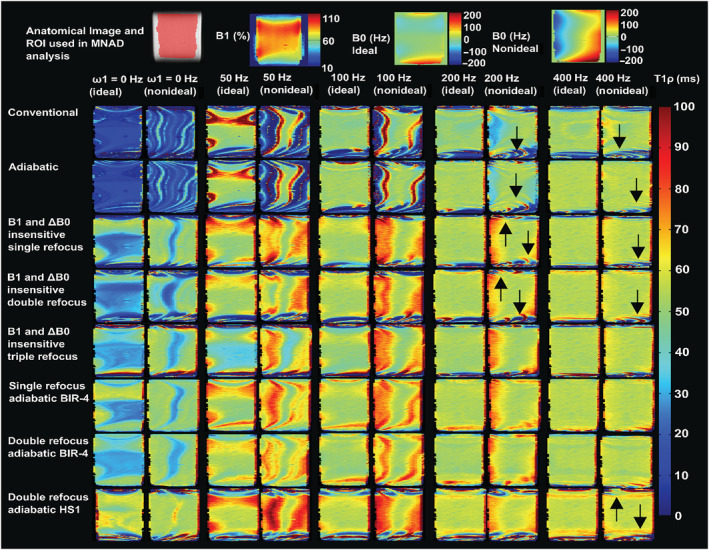
T_1ρ_ relaxation time maps of agar phantom, under as ideal as possible conditions and under non‐ideal conditions, with the sample size covering the region away from the RF center and inhomogeneous B_0_ field, for SL amplitudes of 0–400 Hz acquired with the different methods. Anatomical reference (showing the MNAD analysis ROI with red shading) and the corresponding B_1_ and B_0_ maps are shown at the top. Under the ideal conditions, all the refocused methods provided largely artifact‐free T_1ρ_ relaxation time maps at all SL amplitudes, while the non‐refocused methods showed artifacts at the edges of the FOV at low spin‐lock amplitudes. Under the non‐ideal conditions, the non‐refocused T_1ρ_ methods performed poorly at lower SL amplitudes, while the refocused methods were able to mitigate the most severe artifacts, especially at the higher SL amplitudes. The arrows indicate locations where differences (artifacts) can be noted between the conditions. FOV, field of view; MNAD, mean normalized absolute deviation; RF, radio frequency; ROI, region of interest; SL, spin lock

For the experimental measurements under as ideal as possible conditions, the T_1ρ_ relaxation time maps of the cartilage bone samples, cherry tomatoes, and phantom were visually artifact‐free for all the preparation schemes for SL amplitudes above 100 Hz (Figures 5, 6, 7). Under the non‐ideal conditions, however, at SL amplitudes equal to and below ΔB_0_, the conventional and adiabatic non‐refocused T_1ρ_ relaxation time maps showed banding artifacts (Figures 5, 6, 7). At higher SL amplitudes (ω_1_ > ΔB_0_), the artifacts were significantly suppressed for all methods. For the refocused preparation schemes that compensate for the B_1_ and B_0_ imperfections, the artifacts were significantly reduced even at the lower SL amplitudes. Suppression of artifacts was particularly effective by the preparation schemes utilizing either single or double refocusing with adiabatic BIR‐4 pulses (Figures 5, 6, 7). With the cherry tomato (as well as the phantom), marked banding artifacts are seen at the top and bottom of the samples, where both B_1_ and B_0_ fields deviated from the nominal values, even under the experimentally ideal conditions. At higher spin locking amplitudes, the artifacts due to the non‐homogenous fields were also suppressed in these samples, particularly with the adiabatic BIR‐4 refocusing.

**FIGURE 8:**
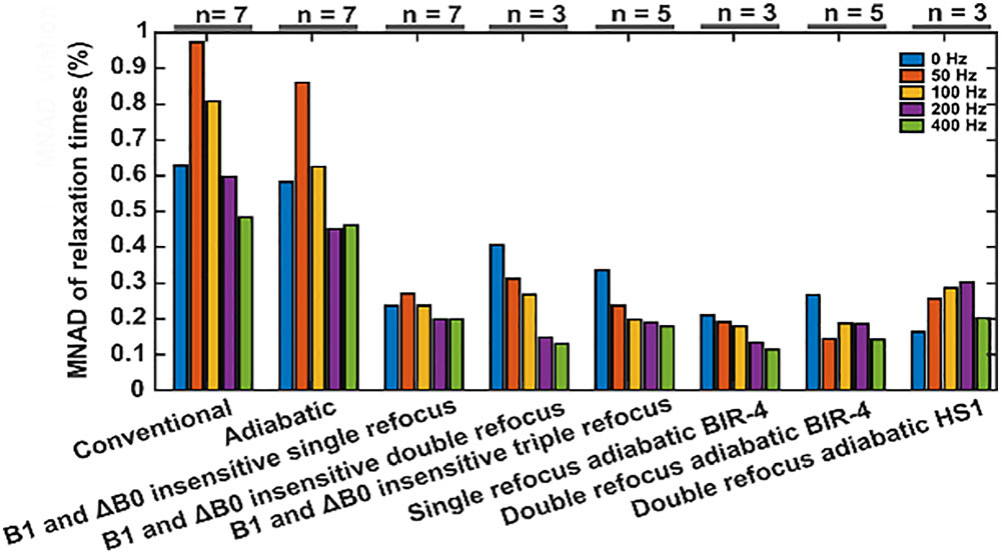
MNAD values of the relaxation times between the ideal and non‐ideal conditions in large ROIs (shown in Figures 5, 6, 7) over all the samples available for each measurement. The refocused preparation schemes stand out by showing the least variation between the ideal and nonideal cases, indicating their robustness against field inhomogeneities regardless of the SL amplitude. MNAD, mean normalized absolute deviation; ROI, region of interest; SL, spin lock

The MNADs within the ROIs were generally the smallest for the refocused schemes (Figure 8), broadly in agreement with the sensitivities observed in the Bloch simulations. Among the refocused hard‐pulse and adiabatic preparation schemes, the double‐ and single‐refocus adiabatic BIR‐4 methods presented the smallest deviations in the experimental data (Figure 8). It should be noted that while all the scanned samples were used in the calculation of the average MNADs, the number of samples was not the same for all preparations because of the later inclusion of some of the preparation schemes in the study (Figure 8).

**FIGURE 9:**
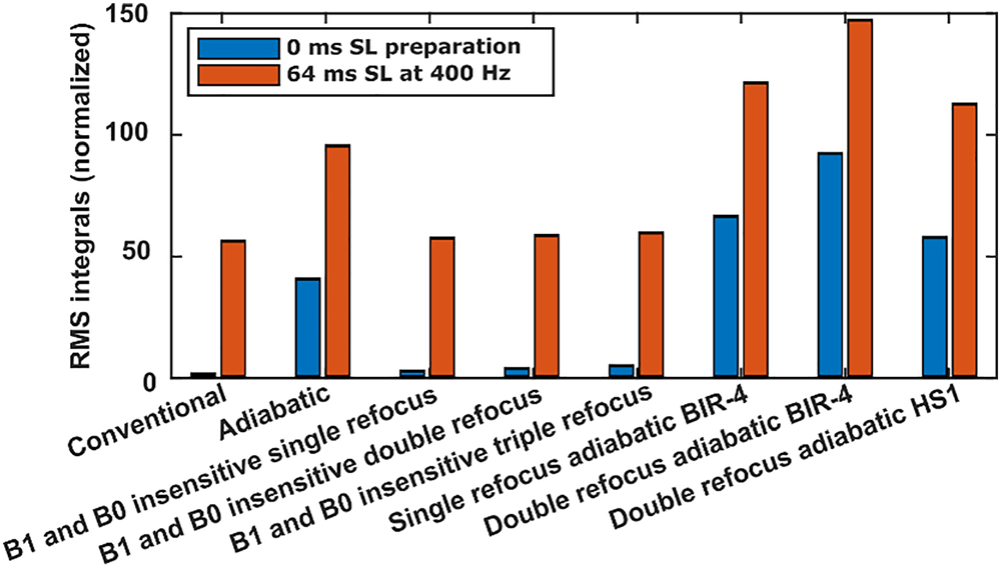
Comparison of the RMS integrals (RF power) of the zero SL duration RF pulse trains (blue bars) and 64‐ms SL duration RF pulse trains at 400‐Hz SL amplitude (orange bars) for the different T_1ρ_ preparation methods normalized to the conventional SL method. All the hard‐pulse methods show negligible difference to the conventional SL method. On the other hand, the adiabatic pulses show significantly increased RF energy deposition. The RMS integrals of the zero SL duration adiabatic pulse schemes are approximately comparable with those of the conventional 64‐ms spin lock at 400 Hz. BIR, B_1_ insensitive rotation; HS, hyperbolic secant; RF, radio frequency; RMS, root mean square; SL, spin lock

The conventional hard‐pulse CW‐T_1ρ_ preparation with only two 90° pulses imposes the least additional RF energy deposition and thus produces the least specific absorption rate (SAR) (Figure 9). The preparations including adiabatic pulses add a constant adiabatic T_1ρ_ weighting in addition to the T2 weighting from finite TE of the readout, and these pulses induce significantly higher RF energy deposition (the RMS integral of the 0‐ms SL pulse for the double‐refocus BIR‐4 is approximately 86 times that of the conventional T_1ρ_ preparation) (Figure 9, Table S1). However, for a plain SL pulse (i.e., without the 90° or 180° pulses) of 50‐ms duration and 400‐Hz amplitude, the RMS integral is ~40 times that of the 0‐ms SL pulse of the conventional T_1ρ_ preparation with the least extra RF. For increasing SL durations and amplitudes, relative differences in the energy deposition between the preparation schemes are reduced (an RMS integral ratio of a SL pulse of 64‐ms duration and 400‐Hz amplitude using double‐refocus BIR‐4 with respect to conventional is reduced from ~86 times to just under three times) (Figure 9, Table S1). The 0‐ms SL adiabatic CW T1ρ pulse, with a longer duration and a reduced maximum RF amplitude of 600 Hz of the AHP, was observed to have approximately one‐quarter of the RMS integral of the original pulse with a maximum amplitude of 2.5 kHz. With the same lower‐power AHP pulses, the RMS integral of a SL pulse of 64‐ms duration and 400‐Hz amplitude was reduced by a factor of approximately 1.5 compared with the original using 2.5‐kHz AHP pulses (Table S1, Figure S6).

## 4 DISCUSSION

T_1ρ_ contrast remains interesting for various applications in the human body because of its sensitivity to low frequency molecular interactions that are often biologically important.^5^, ^9^ The different T_1ρ_ contrast preparation methods, particularly at very low SL amplitude, are however sensitive to imperfections of the imaging field and the RF field. In this study, we proposed four new methods for generating T_1ρ_ contrast and compared them experimentally and numerically with four existing methods for their sensitivity to the field inhomogeneities. The study builds on earlier reports introducing ΔB_0_ and B_1_ insensitive T_1ρ_ preparation schemes,^15^, ^18^, ^19^, ^22^ particularly the one by Witschey et al.,^14^ and utilizes the same theoretical examination of the proposed hard‐pulse schemes (see the supporting information). The results of the study indicate that those methods employing a refocusing pulse are significantly more robust against field inhomogeneities than those methods which do not, and also that combining CW spin locking with fully adiabatic excitation and refocusing is the most robust method against field inhomogeneities. However, the fully adiabatic schemes have the additional cost of significantly increased RF energy deposition. Among the non‐adiabatic hard‐pulse refocusing sequences, the most robust ones were the single‐refocus hard‐pulse CW‐T_1ρ_ method proposed by Witschey et al.^14^ and the proposed triple‐refocus hard‐pulse CW‐T_1ρ_ method. These two outperformed the double‐refocused B‐SL method proposed by Gram et al.,^18^ most likely because this method brings the magnetization back to the positive z axis, while the other two take the magnetization to the negative z axis and are thus more robust against variations in B_0_.

Recently, there has been an increase in interest towards T_1ρ_ dispersion in cartilage,^13^, ^32^, ^33^, ^34^, ^35^, ^36^ because the measurement could provide information beyond a single amplitude T_1ρ_ scan. However, especially lowering the SL amplitudes requires methods that are robust against field inhomogeneities. If the B_0_ variations exceed the spin‐locking amplitude, the locking becomes inefficient, resulting in spurious signal loss, which is further amplified with methods that do not compensate for field variations.^1^, ^12^

The theoretical considerations regarding the triple‐refocused hard‐pulse CW‐T_1ρ_ preparation lead to the same conclusions that were found for the single‐refocused preparation scheme earlier by Witschey et al.,^14^ suggesting the methods should be approximately equal. The simulations showed a slightly broader flat response with respect to variations in B_0_ for the single‐refocus method, while the response of the triple‐refocused method was slightly smoother. The double‐refocused pulse scheme brings the magnetization back to the positive z axis; however, it appears to require nearly perfect 90° and 180° pulses, while the single‐ and triple‐refocused methods only require that the 180° pulses should be nearly perfect. Because of this difference, the single‐ or triple‐refocused schemes appeared more robust against field inhomogeneities, as confirmed by the simulations. In practice, however, all the refocused hard‐pulse options were observed to be very similar in soft tissues.

Adiabatic pulses are known for their excellent tolerance to RF inhomogeneity^28^ and thus stand out as an interesting possibility to improve the robustness of CW T_1ρ_ preparation. Furthermore, adiabatic T_1ρ_ could be measured in fully adiabatic mode, using a train of AFP HS RF pulses, instead of a constant amplitude CW SL pulse in between AHP pulses.^22^, ^24^, ^29^, ^37^, ^38^ In comparison with a CW SL with fixed B1 amplitude and orientation, the adiabatic T_1ρ_ SL varies between off‐resonance and on‐resonance T1ρ during the adiabatic sweep, where the amplitude and frequency of the pulse are modulated during the time course of the pulse.^39^ From the simulations, it was evident that the refocused adiabatic methods presented here are highly insensitive to ΔB_0_ and B_1_ field inhomogeneities. The robustness of the refocused adiabatic methods exceeded the simulated range of variation for the RF power, while the robustness against B_0_ variations depended on the specific scheme. The double‐refocused adiabatic BIR‐4 and HS1 versions were found to be the most robust in the simulations, while experimentally, the double‐refocused BIR‐4 scheme was found to be the most robust. The low‐powered (600‐Hz) adiabatic CW‐T_1ρ_, which had an AHP pulse approximately four times longer than the high‐powered (2.5‐kHz) AHP pulse, was highly insensitive to field inhomogeneities at the higher SL amplitude of 400 Hz in the simulations (Figure S3B). This simulation demonstrates that when the maximum B_1_ amplitude of the AHP pulses is brought closer to the spin‐locking amplitude, then adiabatic CW‐T_1ρ_ becomes highly insensitive to B_0_ inhomogeneities that are of the order of or smaller than the spin‐locking amplitude.

The experimental findings under the ideal and non‐ideal conditions largely confirmed the observations of the simulations. The non‐refocused conventional and adiabatic schemes under the non‐ideal conditions fell behind all the other schemes that utilized refocusing, although under the ideal conditions, the relaxation time maps were mostly clean, particularly at higher SL amplitudes. The ΔB_0_ and B_1_ insensitive single‐refocus method^14^ and the proposed triple‐refocus method were experimentally approximately equal, while the B‐SL method^18^ exhibited poorer performance and more banding artifacts, especially at lower SL amplitudes. On the other hand, the adiabatic BIR‐4 refocused schemes produced very clean T_1ρ_ maps compared with all other methods in all specimens, suppressing the most artifacts (Figure S4). The non‐refocused schemes showed severe banding artifacts in the T_1ρ_ relaxation time maps under the non‐ideal conditions, at SL amplitudes equal to and below ΔB_0_. At higher SL amplitudes (ω_1_ > ΔB_0_, or ω_1_>> ΔB_0_), the banding artifacts were minimal for all the schemes, unless B_1_ variation was also present.

The differences in the sensitivities to field inhomogeneities between the preparation schemes were assessed by calculating the MNAD values between the measurements conducted at ideal versus non‐ideal conditions. This approach, while potentially dependent on the changes in the experimental conditions, provides a handle on the sensitivities of the methods, summarizing the results over all the measured samples. Among the hard‐pulse schemes, the non‐refocused preparations stood out with the largest deviations between the ideal and non‐ideal cases, while the refocused methods showed significantly smaller deviation between the cases at all SL amplitudes. The adiabatic refocused schemes were aligned with the hard‐pulse alternatives with similar small deviations. However, these analyses were conducted only in the tissues that had high SNR and were not clearly at off‐resonance (such as the fatty bone marrow tissue). Further experimental differences were seen at the extreme areas, such as the fat, or the edges of the coil‐visible region for the tomato specimen in Figures 5 and 6, and particularly in the phantom (Figure 7), where the non‐refocused methods, the B‐SL method,^18^ and the double‐refocus adiabatic HS1 preparations showed signal loss and banding artifacts. The experimental performance of the adiabatic double‐refocus scheme incorporating HS1 inversion pulses was not as good as that of the BIR‐4 approach, despite providing the most promising simulation results. This could be because of the flip angle dispersion effects of the HS1‐AFP pulse on the magnetization components not being collinear with it,^28^ as is the case here. Two HS1‐pulses were utilized to compensate for this effect, but the result remained inferior to that achieved by using an adiabatic plane rotation BIR‐4 pulse.

In the clinical setting, T_1ρ_ relaxation measurements could provide important insights into disease diagnosis and progression.^33^, ^40^, ^41^, ^42^ However, small B_0_ and B_1_ variations are inevitable and interfere with the T_1ρ_ quantification. This is particularly the case for low amplitude spin locking, because the threshold of B_0_ variation affecting measurements depends on the spin‐lockin amplitude. The double‐refocus adiabatic BIR‐4 scheme was found to be the most robust against field inhomogeneities for improving the T_1ρ_ quantification. However, the most significant problem with this method is its significantly increased RF energy deposition: as realized here, the baseline zero SL pulse has a duration of approximately 18 ms at an RMS amplitude of 2.3 kHz, which is already well beyond what is typically even achievable on a clinical scanner (often the maximum transmit power is below 1 kHz, even for local transmit coils).^43^ Besides the increased power requirements, such pulses are also likely to exceed SAR safety limits,^14^ further limiting the use of such T_1ρ_ preparations. Among the less RF‐intensive, yet ΔB_0_ and B_1_ insensitive T_1ρ_ preparation schemes, the single‐refocus scheme^14^ with minimal RF energy deposition appears to be the most feasible for in vivo imaging. However, because the magnetization after this preparation will be at the negative z axis, a spin‐echo type of readout sequence would be preferable over a gradient‐echo sequence with relatively small tip angles, which will drive the magnetization through zero if longer echo trains are collected. Alternatively, for a gradient‐echo readout sequence, an additional (adiabatic) inversion pulse could potentially be utilized at the end of the preparation to avoid this effect. Considering the overall scan duration, gradient‐echo sequences with short TR and RF cycling^44^ or tailored flip angles^45^ could be utilized to enable faster scans.

Other possibilities for improved T_1ρ_ have been presented previously, such as the one by Mitrea et al.^15^ Initial tests (Figure S5), however, suggested it to be more sensitive to field variations than the single‐refocus method reported by Witschey et al.,^14^ further supported by the simulations (see the supporting information). Another very promising approach utilizes adiabatic excitation and rewinder pulses at the same amplitude as the target SL amplitude.^16^, ^46^, ^47^ Simulations with a nearly matched amplitude SL pulse^16^, ^47^ suggested that this non‐refocused adiabatic scheme performs very well against the field inhomogeneities (see the supporting information). However, this sequence is more akin to the adiabatic T_1ρ_ method,^7^, ^24^, ^25^, ^43^ and is a combination of on‐resonance and off‐resonance T_1ρ_ relaxation. Another potential challenge with this method is maintaining the adiabatic condition at very low SL amplitudes. Utilizing fully adiabatic spin locking^22^, ^24^, ^29^, ^37^, ^38^ can further mitigate the effects of field inhomogeneities and even provide slice selectivity^37^ as well as reduced orientation/magic angle dependence.^7^ A variation of the double‐refocused hard‐pulse preparation scheme investigated here^18^ was presented recently with promising results, but without direct comparison with other preparation methods.^48^ Besides presenting a method for faster T_1ρ_ acquisition by using tailored variable flip angle scheduling, Johnson et al.^45^ also utilized a partially adiabatic variation of the single‐refocus method by Witschey et al.,^14^ replacing the hard 90° pulses with adiabatic pulses. This variation presents another interesting option for T_1ρ_ preparation; however, no direct comparison with other T_1ρ_ preparations with respect to sensitivity to inhomogeneities was provided.

The present study has certain limitations, including a limited selection of previously presented methods for the experimental generation of T_1ρ_ contrast. The number of samples is limited, and all the experiments were carried out at 9.4 T and using a relatively high maximum B_1_ amplitude. However, the differences between the methods were generally confirmed with the simulations; similar practical differences may be expected with B_0_ and B_1_ variations regardless of the main field strength, although the practical in vivo importance is ultimately revealed with real measurements.

In conclusion, artifacts arising from the field inhomogeneities in CW‐T_1ρ_–weighted imaging can be efficiently suppressed by different refocused spin‐locking pulse schemes. In this numerical, experimental, and theoretical comparison of different T_1ρ_ contrast preparation methods, the double‐refocus adiabatic BIR‐4 preparation was found to be the most robust. However, because of the excessive RF energy deposition of the adiabatic method, its use is likely restricted to the preclinical setting. Of the less RF‐intensive methods, the ΔB_0_ and B_1_ compensated single‐refocus hard‐pulse CW‐T_1ρ_ method reported by Witschey et al.^14^ and the proposed triple‐refocused method proved to be very robust against field inhomogeneities. The simulations confirm the increased robustness of the low‐power AHP CW spin locking, and both the experimental and the simulation findings promote the use of the previously reported hard‐pulse single‐refocus ΔB_0_ and B_1_ insensitive method for clinical use, while the adiabatic double‐refocused BIR‐4 method could be preferred for ex vivo experiments.

## CONFLICT OF INTERESTS

The authors declare no conflicts of interest.

## Supporting information


**Figure S1:** Triple refocus magnetization path under on‐resonance conditions. The magnetization (M[0‐]) is flipped from the longitudinal axis (z‐axis) with an angle α, which does not need to be 90°, towards the transverse plane (y‐axis) (M[0+]) and nutates about the z_1_” axis (M[τ−/4]) and at time τ/4 is flipped by 2α about y‐axis (M[τ+/4]) and nutates (M[τ−/2]) around z_2_”, this trend of rotation and nutation is the same for the rest of the pulses in the train. The final magnetization (M[τ‐], after full spin‐locking) is brought to the negative longitudinal axis (M[τ+]) by another flip angle α. Artifacts due to imperfect 180° are compensated to an extent by the multiple refocuses.
**Figure S2:** Field insensitive spin lock with double refocusing for T1ρ relaxation measurements. Magnetization path under on‐resonance conditions. The magnetization (M[0‐]) is flipped from the longitudinal plane (z‐axis) with an angle α, which does not need to be 90° towards the transverse plane (y‐axis) (M[0+]) and nutates about the z1” axis (M[τ−/4]) and at time τ+/4 is flipped by 2α about y‐axis (M[τ+/4]) and nutates (M[τ‐3/4]) around z2”, this trend of rotation and nutation is same for the rest of the pulses in the train. The final magnetization (M [τ‐], after full spin‐locking) returns to the positive longitudinal axis (M [τ+]) with another flip angle ‐α. Artifacts due to imperfect 180° are compensated to an extent by the double refocusing.
**Figure S3:** Simulations for *PSC‐SL* (a) proposed by Mitrea et al^1^, and conventional adiabatic sequence with reduced maximum power of the AHP pulse to 600 Hz (b) with the same ΔB_0_ and B_1_ field inhomogeneity ranges of up to ±1 kHz and ±40% as presented in this manuscript, for spin‐lock durations of 32 ms and 128 ms, and for spin‐lock amplitudes of 100 Hz and 400 Hz.
**Figure S4**. Relative differences between the T1ρ maps acquired under the ideal and non‐ideal conditions for the worst performing conventional (Figure 1A), and for the two best performing, single‐refocus hard pulse (Figure 1B) and double‐refocus adiabatic BIR‐4 (Figure 2C) preparation schemes at spin‐lock amplitudes ranging from 0 to 400 Hz.
**Figure S5**. T1ρ relaxation time maps for paired self‐compensated single refocus PSC‐SL sequence proposed by Mitrea et al^1^, under the non‐ideal conditions for spin‐lock amplitudes of 0–400 Hz. Anatomical reference and the corresponding B_0_ map are shown at the top.
**Figure S6** c. Comparison of the RMS integrals (RF power) of the zero spin‐lock duration RF pulse trains (blue bars) and 64 ms spin‐lock duration RF pulse trains at 400 Hz spin‐lock amplitude (orange bars) of the conventional pulse, paired self‐compensated PSC‐SL pulse sequence proposed by Mitrea et al^1^, and adiabatic CW pulse with 600 Hz maximum AHP power T_1ρ_ preparation methods, normalized to the RMS integral of the zero spin‐lock duration conventional spin‐lock method.
**Figure S7.** Calculated approximate flat widths for the Bloch simulations of all the sequences in Figures 1 and 2 over ΔB_0_ (±1 kHz) at the correct B_1_ amplitude. Simulations were run with spin‐lock durations ranging from τ = 0 to 256 ms in 2 ms steps, and with spin‐lock amplitudes from 0 to 1,000 Hz in 10 Hz steps.
**Figure S8.** Calculated approximate flat widths for the Bloch simulations of all the sequences in Figures 1 and 2 over ΔB_1_ (±40%) at the correct B_0_ amplitude. Simulations were run with spin‐lock durations ranging from τ = 0 to 256 ms in 2 ms steps, and with spin‐lock amplitudes from 0 to 1,000 Hz in 10 Hz steps.
**Figure S9:** Calculated approximate flat widths for the Bloch simulations of the *PSC‐SL* proposed by Mitrea et al^1^, and for the adiabatic CW pulse with 600 Hz maximum AHP power T_1ρ_ preparation methods with similar ΔB_0_ and B_1_ field inhomogeneities of up to ±1 kHz and ±40% as presented in this manuscript. Simulations were run with spin‐lock durations ranging from τ = 0 to 256 ms in 2 ms steps, and with spin‐lock amplitudes from 0 to 1,000 Hz in 10 Hz steps.
**Table S1:** Pulse length, RMS integral and RMS amplitude values of preparation schemes with the zero spin‐lock (SL) pulses and with 64 ms SL at 400 Hz amplitude pulses.

## Data Availability

All the data of the study are openly available at the Zenodo archives at http://doi.org/10.5281/zenodo.5547762.
