# Supplementary material for: New methods for robust continuous wave T1ρ relaxation preparation
Source: NMR Biomed. 2022 Oct 7;36(2):e4834. doi: 10.1002/nbm.4834 (PMC10078184; doi:10.1002/nbm.4834)
Supplement: Supplementary file 1 — Figure S1: Triple refocus magnetization path under on‐resonance conditions. The magnetization (M[0‐]) is flipped from the longitudinal axis (z‐axis) with an angle α, which does not need to be 90°, towards the transverse plane (y‐axis) (M[0+]) and nutates about the z1” axis (M[τ−/4]) and at time τ/4 is flipped by 2α about y‐axis (M[τ+/4]) and nutates (M[τ−/2]) around z2”, this trend of rotation and nutation is the same for the rest of the pulses in the train. The final magnetization (M[τ‐], after full spin‐locking) is brought to the negative longitudinal axis (M[τ+]) by another flip angle α. Artifacts due to imperfect 180° are compensated to an extent by the multiple refocuses. Figure S2: Field insensitive spin lock with double refocusing for T1ρ relaxation measurements. Magnetization path under on‐resonance conditions. The magnetization (M[0‐]) is flipped from the longitudinal plane (z‐axis) with an angle α, which does not need to be 90° towards the transverse plane (y‐axis) (M[0+]) and nutates about the z1” axis (M[τ−/4]) and at time τ+/4 is flipped by 2α about y‐axis (M[τ+/4]) and nutates (M[τ‐3/4]) around z2”, this trend of rotation and nutation is same for the rest of the pulses in the train. The final magnetization (M [τ‐], after full spin‐locking) returns to the positive longitudinal axis (M [τ+]) with another flip angle ‐α. Artifacts due to imperfect 180° are compensated to an extent by the double refocusing. Figure S3: Simulations for PSC‐SL (a) proposed by Mitrea et al1, and conventional adiabatic sequence with reduced maximum power of the AHP pulse to 600 Hz (b) with the same ΔB0 and B1 field inhomogeneity ranges of up to ±1 kHz and ±40% as presented in this manuscript, for spin‐lock durations of 32 ms and 128 ms, and for spin‐lock amplitudes of 100 Hz and 400 Hz. Figure S4. Relative differences between the T1ρ maps acquired under the ideal and non‐ideal conditions for the worst performing conventional (Figure 1A), and for the two best performing, single‐r [file NBM-36-0-s001.docx]

**New methods for robust continuous wave T_1ρ_ relaxation preparation**

Pala S^1^ (MSc), Hänninen NE^1,2^ (MSc), Nykänen O^1,2^ (PhD), Liimatainen T^2,3^ (PhD), Nissi MJ^1,2*^(PhD)

^1^Department of Applied Physics, University of Eastern Finland, Kuopio, Finland

^2^Research Unit of Medical Imaging, Physics and Technology, University of Oulu, Oulu, Finland

^3^Department of Radiology, Oulu University Hospital, Oulu, Finland

**Supplementary Information**

**Theory**

*Hard pulse CW-T1ρ preparation scheme:*

Here we focus on the single-refocused^1^, double-refocused^2^ and on novel triple-refocused hard pulse CW-T1ρ preparation schemes (Fig. 1C and 1D, Supplementary Fig. S1, S2 and S3). The analysis follows the notation of Witschey et al^3^. For the derivations of the sensitivities of the single-refocused and the conventional non-refocused T1ρ preparation schemes investigated here (Table 1 and Fig. 1A-B), the reader is referred to the previous publication^3^. The double-refocused preparation (Fig. 1C) investigated here is the B-SL preparation reported by Gram et al^2^, but with a 90° phase-shift in the notation of the B_1_ phases.

***Triple refocused ΔB0 and B1 insensitive hard pulse spin lock* (Fig. 1D & S1)**

*90x -τ/4y -180y - τ/4(-y) - 180y - τ/4y -180(-y) - τ/4(-y) -90x,*

Where *τ* represents duration of the spin-locking and *x* and *y* the phases of the RF pulses (Fig. 1D).

Bloch equations in the rotating frame of reference describe the behavior of the magnetization over time. The vector representation of the magnetization M at the spatial location r = [x, y, z] and time *t* during spin locking sequence is given by

M(r; *t*) = [*Mx* (r; *t*), *My* (r; *t*), *Mz* (r; *t*)] ^T^ (1)

Magnetization vector before excitation in the rotating frame is

M(r; 0^-^ ) = [0, 0, *M_0_*(r; 0^-^)]^T^  (2)

where *M_0_* denotes the initial magnetization or equilibrium magnetization and *t* = 0^-^ is the time prior to the excitation and T denotes the transpose operation. Excluding the effects of relaxation, the matrix representation of the external RF pulse applied to flip the magnetization is denoted as *R*_θ_(α), where the rotation is denoted by *R*, the phase of the pulse by θ, and the flip angle by α. For each of the pulse sequences in Fig. 1, *R_x_* (90°) denotes the initial rotation matrix that ideally produces a 90° flip angle around *x*-axis in the rotating frame of reference. Practically, the variations in the RF field strength *B*_1_ may produce imperfect 90° flip angles, resulting instead in flip angles of α, given by

α(r) = γB_1_(r) *τ* (3)

Considering the rotation matrices:

R_x_ (α) = $\left[ \begin{matrix} 1 & 0 & 0 \\ 0 & cos(\alpha) & sin(\alpha) \\ 0 & -sin(\alpha) & cos(\alpha) \end{matrix} \right]$ R_y_ (α) = $\left[ \begin{matrix} cos(\alpha) & 0 & -sin(\alpha) \\ 0 & 1 & 0 \\ sin(\alpha) & 0 & cos(\alpha) \end{matrix} \right]$

*R_z_ (α) =* $\left[ \begin{matrix} cos(\alpha) & sin(\alpha) & 0 \\ -sin(\alpha) & cos(\alpha) & 0 \\ 0 & 0 & 1 \end{matrix} \right]$ (4)

The magnetization after the excitation, at the time *t* = 0^+^, can be written as

M(0^+^) = *R*_θ_(α) M(0^-^) = $\left[ \begin{matrix} 0 \\ M_{0}sin(\alpha) \\ M_{0}cos(\alpha) \end{matrix} \right]$ (optimally M(0^+^) = *R*_x_(90°) M(0^-^)), (5)

where the spatial dependence on r is taken implicitly for the rest of the derivation. After tilting the magnetization to the xy-plane, it is locked in the transverse plane by the spin-lock RF pulse for the time *τ/4,* during which the magnetization nutates about the effective field *z_1_*’’, which is at an angle φ from the longitudinal axis (z-axis) (Fig. S1):

φ = tan^-1^(ω_1_ / Δω_0_), (6)

where Δω_0_ = ω_0_ – ω_RF_. Nutation around the effective field under off-resonance in matrix notation is given by R_z’’_ (ω_eff_𝜏), where |ω_eff_| = $\sqrt{\omega_{1}^{2}+\Delta\omega_{0}^{2}}$. Under the off-resonance conditions, the rotation of the magnetization can be described using a tilted frame of reference given by

R_z’’_ (ω_eff_𝜏) = R_x_ (φ) R_z_ (ω_eff_𝜏) R_x_ (-φ) (7)

After the first part of spin-locking, the first refocusing pulse *R*_y_(180°) is applied, due to which the magnetization is flipped, followed by a spin lock for another *τ/4,* during which the magnetization nutates around *z_2_’’* (Fig. S1)*.* The magnetization then experiences the rest of the refocusing and spin-locking pulses along their respective orientations as described above. Finally, the magnetization is flipped back to the longitudinal axis (to the negative z-axis in the case of the triple refocused spin-locking scheme presented here) by another 90° pulse. The final magnetization after all the rotations by the pulse train, including all the spin-lock pulses is given by:

M(*τ* ^+^) = *R_x_* (α) R_x_ (-φ)R*z_2_’’*(ω_eff_𝜏/4)R_x_ (φ) *R_-y_* (2α)

x R_x_ (φ)R*z_1_’’*(ω_eff_𝜏/4)R_x_ (-φ) *R_y_* (2α)

x R_x_ (-φ)R*z_2_’’*(ω_eff_𝜏/4)R_x_ (φ) *R_y_* (2α)

x R_x_ (φ)R*z_1_’’*(ω_eff_𝜏/4)R_x_ (-φ) *R_x_* (α) M(0^-^) (8)

If ω_1_ >> Δω_0_ and φ = 90°, equation (8) reduces to:

M(𝜏 ^+^) = *R_x_* (α) R _-y_ (ω_1_𝜏/4) *R-_y_* (2α) R_y_ (ω_1_𝜏/4) *R_y_* (2α)

x R _-y_ (ω_1_𝜏/4) *R_y_* (2α) *R*_y_ (ω_1_𝜏/4) *R_x_* (α) M(0^-^) (9)

In matrix form, the equation (9), can be written as

M($\tau$ ^+^) = $\left[ \begin{matrix} 1 & 0 & 0 \\ 0 & cos(\alpha) & sin(\alpha) \\ 0 & -sin(\alpha) & cos(\alpha) \end{matrix} \right]$ x $\left[ \begin{matrix} cos(\omega_{eff}\tau/4) & 0 & -sin(\omega_{eff}\tau/4) \\ 0 & 1 & 0 \\ sin(\omega_{eff}\tau/4) & 0 & cos(\omega_{eff}\tau/4) \end{matrix} \right]$

x $\left[ \begin{matrix} cos(2\alpha) & 0 & -sin(2\alpha) \\ 0 & 1 & 0 \\ sin(2\alpha) & 0 & cos(2\alpha) \end{matrix} \right]$ x $\left[ \begin{matrix} cos(\omega_{eff}\tau/4) & 0 & sin(\omega_{eff}\tau/4) \\ 0 & 1 & 0 \\ -sin(\omega_{eff}\tau/4) & 0 & cos(\omega_{eff}\tau/4) \end{matrix} \right]$ x $\left[ \begin{matrix} cos(2\alpha) & 0 & -sin(2\alpha) \\ 0 & 1 & 0 \\ sin(2\alpha) & 0 & cos(2\alpha) \end{matrix} \right]$ x $\left[ \begin{matrix} cos(\omega_{eff}\tau/4) & 0 & -sin(\omega_{eff}\tau/4) \\ 0 & 1 & 0 \\ sin(\omega_{eff}\tau/4) & 0 & cos(\omega_{eff}\tau/4) \end{matrix} \right]$

x $\left[ \begin{matrix} cos(2\alpha) & 0 & sin(2\alpha) \\ 0 & 1 & 0 \\ -sin(2\alpha) & 0 & cos(2\alpha) \end{matrix} \right]$ x $\left[ \begin{matrix} cos(\omega_{eff}\tau/4) & 0 & sin(\omega_{eff}\tau/4) \\ 0 & 1 & 0 \\ -sin(\omega_{eff}\tau/4) & 0 & cos(\omega_{eff}\tau/4) \end{matrix} \right]$

x $\left[ \begin{matrix} 0 \\ M_{0}sin(\alpha) \\ M_{0}cos(\alpha) \end{matrix} \right]$

The residual transverse magnetization is eliminated by the spoiler gradients; thus, the final magnetization M_z_ (𝜏^+^) under condition (ω_1_ >> Δω_0_) from equation (9) becomes (here we particularly consider 2α and α as separate entities and keep them deliberately separated):

M_z_($\tau$ ^+^) = $M_{0}$ [$\frac{1}{2}$(cos(2 * α) + cos(2α) + cos(2 * α) cos(2α) – 1)], (10)

= $M_{0}$ [$\frac{1}{2}$(cos^2^(α) - sin^2^(α) + cos(2α) + (cos^2^(α) - sin^2^(α)) cos(2α) – 1)] (11)

When the initial flip *R_x_* (α) is not 90°, the magnetization is influenced by the 2α. While if, *R_x_* (α) is 90°, the magnetization is unaffected by the pulse and is reduced to -M(0^-^).

Conversely, if ω_1_ << Δω_0,_ the angle φ between the z’’ and z is nearly 0° and the equation (8) is reduced to

M (𝜏^+^) = *R_x_* (α) R _z_ (Δω_0_ 𝜏/4) *R_-y_* (2α) R_z_ (Δω_0_ 𝜏/4) *R_y_* (2α)

x R _z_ (Δω_0_ 𝜏/4) *R_y_*(2α) R_z_ (Δω_0_ 𝜏/4) *R_x_* (α) M(0^-^) (12)

Equation (12) in matrix notation can be written as,

M($\tau$ ^+^) = $\left[ \begin{matrix} 1 & 0 & 0 \\ 0 & cos(\alpha) & sin(\alpha) \\ 0 & -sin(\alpha) & cos(\alpha) \end{matrix} \right]$ x $\left[ \begin{matrix} cos(\omega_{eff}\tau/4) & sin(\omega_{eff}\tau/4) & 0 \\ -sin(\omega_{eff}\tau/4) & cos(\omega_{eff}\tau/4) & 0 \\ 0 & 0 & 1 \end{matrix} \right]$

x $\left[ \begin{matrix} cos(2\alpha) & 0 & -sin(2\alpha) \\ 0 & 1 & 0 \\ sin(2\alpha) & 0 & cos(2\alpha) \end{matrix} \right]$ x $\left[ \begin{matrix} cos(\omega_{eff}\tau/4) & sin(\omega_{eff}\tau/4) & 0 \\ -sin(\omega_{eff}\tau/4) & cos(\omega_{eff}\tau/4) & 0 \\ 0 & 0 & 1 \end{matrix} \right]$ x $\left[ \begin{matrix} cos(2\alpha) & 0 & -sin(2\alpha) \\ 0 & 1 & 0 \\ sin(2\alpha) & 0 & cos(2\alpha) \end{matrix} \right]$ x $\left[ \begin{matrix} cos(\omega_{eff}\tau/4) & sin(\omega_{eff}\tau/4) & 0 \\ -sin(\omega_{eff}\tau/4) & cos(\omega_{eff}\tau/4) & 0 \\ 0 & 0 & 1 \end{matrix} \right]$ x $\left[ \begin{matrix} cos(2\alpha) & 0 & sin(2\alpha) \\ 0 & 1 & 0 \\ -sin(2\alpha) & 0 & cos(2\alpha) \end{matrix} \right]$ x $\left[ \begin{matrix} cos(\omega_{eff}\tau/4) & sin(\omega_{eff}\tau/4) & 0 \\ -sin(\omega_{eff}\tau/4) & cos(\omega_{eff}\tau/4) & 0 \\ 0 & 0 & 1 \end{matrix} \right]$

x $\left[ \begin{matrix} 0 \\ M_{0}sin(\alpha) \\ M_{0}cos(\alpha) \end{matrix} \right]$

Specifically, if *R_y_* (2α) is *R_y_* (180°), then the final magnetization from the equation (12) is reduced to

M_z_ (𝜏^+^) = -M(0^-^) (13)

and is independent of off-resonance effects. At intermediate field strengths (ω_1_ ∼ Δω_0_), however this scheme requires both *R_x_* (α) = *R_x_* (90°) and *R_y_* (2α) = *R_y_* (180°) to remove the dependence of the final magnetization on cos(ω_1_𝜏/4) terms. In practice, the inability to achieve perfect 180° flip is partially compensated by the additional refocusing pulses in the triple-refocused spin-lock scheme.

***Double-refocused ΔB0 and B1 insensitive hard pulse train***^2^ **(Fig. 3C, Fig. S2):**

*90_x_ -τ/4_y_ -180_y_ - τ/2_-y_ – 180_-y_ - τ/4_y_ -90_-x_*

The magnetization expression for the full pulse scheme is

M($\tau$ ^+^) = *R-_x_* (α) R_x_ ($\varphi$)R$z_{1}^{''}$ ($\omega_{eff}\tau/4$)R_x_ (-$\varphi$) *R-_y_* (2α)

x R_x_ ($-\varphi$)R$z_{2}^{''}$ ($\omega_{eff}\tau/2$)R_x_ ($\varphi$) *R_y_* (2α)

x R_x_ ($\varphi$)R$z_{1}^{''}$ ($\omega_{eff}\tau/4$)R_x_ ($-\varphi$) *R_x_* (α) M(0^-^) (14)

If $\omega_{1}\gg$ $\Delta\omega_{0}$ and $\varphi$ = 90°, the equation (14) reduces to,

M($\tau$ ^+^) = *R_-x_* (α) R_y_ ($\omega_{1}\tau/4$) *R_-y_* (2α) R _-y_ ($\omega_{1}\tau/2$) *R_y_* (2α).

R_y_ ($\omega_{1}\tau/4$) *R_x_* (α) M(0^-^) (15)

and the final longitudinal magnetization is reduced to

M_z_ ($\tau$ ^+^) = M(0^-^) (16)

The resulting magnetization is unaffected by the spin-lock nutation and α terms.

Conversely, if $\omega_{1}\ll$ $\Delta\omega_{0}$, the angle $\varphi$ between the z’’ and z is nearly 0°, and equation (14) is reduced to

M($\tau$ ^+^) = *R_-x_* (α) R_z_ ($\Delta\omega_{0}\tau/4$) *R_-y_* (2α) R_z_ ($\Delta\omega_{0}\tau/2$) *R_y_* (2α).

x R_z_ ($\Delta\omega_{0}\tau/4$) *R_x_* (α) M(0^-^) (17)

M_z_ ($\tau$ ^+^) = M(0^-^) [cos(2*2α) + 8cos^2^($\frac{2\alpha}{2}$) cos^2^($\frac{\Delta\omega_{0}\tau}{4}$) - 16 cos^4^($\frac{2\alpha}{2}$) cos^2^($\frac{\Delta\omega_{0}\tau}{4}$)+ 8 cos^4^($\frac{2\alpha}{2}$) cos^4^($\frac{\Delta\omega_{0}\tau}{4}$)+ 8 cos^2^(α) cos^4^($\frac{2\alpha}{2}$) cos^2^($\frac{\Delta\omega_{0}\tau}{4}$)- 8 cos^2^(α) cos^4^($\frac{2\alpha}{2}$) cos^4^($\frac{\Delta\omega_{0}\tau}{4}$) ]

If *R_x_* (α) is not *R_x_* (90°), the magnetization is influenced by the $cos({\Delta\omega}_{0}\frac{\tau}{4}$) term and the 2α term, while if *R_x_* (α) is *R_x_* (90°) the magnetization is influenced only by the 2α term and is independent of B_0_ fluctuations. Furthermore, specifically if *R_y_* (2α) is *R_y_* (180°), i.e., assuming cos(2*2α) = cos(360°) and cos($\frac{2\alpha}{2}$) = cos(90°), the equation (15) reduces to

M_z_($\tau$ ^+^) = M(0^-^) (18)

and is independent of the off-resonance effects$.$ However, at intermediate field strengths $\omega_{1}\sim$ $\Delta\omega_{0}$ the sequence demands both *R_±x_* (α) to be *R_±x_* (90°) and *R_y_* (2α) to be *R_y_* (180°) to remove the effective field terms from the equation. The double refocused spin-locking scheme, B-SL presented by Gram et al^2^, is the same as the sequence presented here, but with difference of 90° in the phases of the B1.

$\boldsymbol{\Delta}\boldsymbol{B}_{\boldsymbol{0}}$ ***and B_1_ insensitive single refocus spin lock pulse:* *PSC-SL*** ***scheme*** ^1^**:**

*90_x_ -τ/4_y_ - τ/4_-y_ - 180_y_ - τ/4_y_ - τ/4_-y_ -90_-x_*

The final longitudinal magnetization of the pulse under condition $\omega_{1}\gg$ $\Delta\omega_{0}$ can be reduced to

M_z_($\tau$ ^+^) = M(0^-^) [cos^2^(α) cos(2α) + sin^2^(α)] (19)

This equation (19) is similar to that derived for the scheme introduced by Zeng et al^4^. If *R_x_* (α) is not *R_x_* (90°), the magnetization is dependent on the 2α term. Conversely, under condition $\omega_{1}\ll$ $\Delta\omega_{0}$, the final magnetization becomes

M_z_($\tau$ ^+^) = M(0^-^) [cos^2^(α) -1 + 2sin^2^(α) sin^4^($\Delta\omega_{0}\frac{\tau}{4}$) + cos^2^(α) cos(2α) + 2sin^2^(α) cos^4^($\Delta\omega_{0}\frac{\tau}{4}$) - 4sin^2^(α) cos(2α) cos^2^($\Delta\omega_{0}\frac{\tau}{4}$) sin^2^($\Delta\omega_{0}\frac{\tau}{4}$) - 4sin(α) cos(α) sin(2α) cos($\Delta\omega_{0}\frac{\tau}{4}$) sin($\Delta\omega_{0}\frac{\tau}{4})$] (20)

Specifically, if *R_x_* (2α) is *R_x_* (180°), equation (20) reduces to

M_z_($\tau$ ^+^) = M(0^-^)

At intermediate strengths this pulse trains requires *R_x_*(α) to be a perfect 90° and *R_y_*(2α) to be 180° to get rid of the nutation terms.

**Supplementary Figures**

**
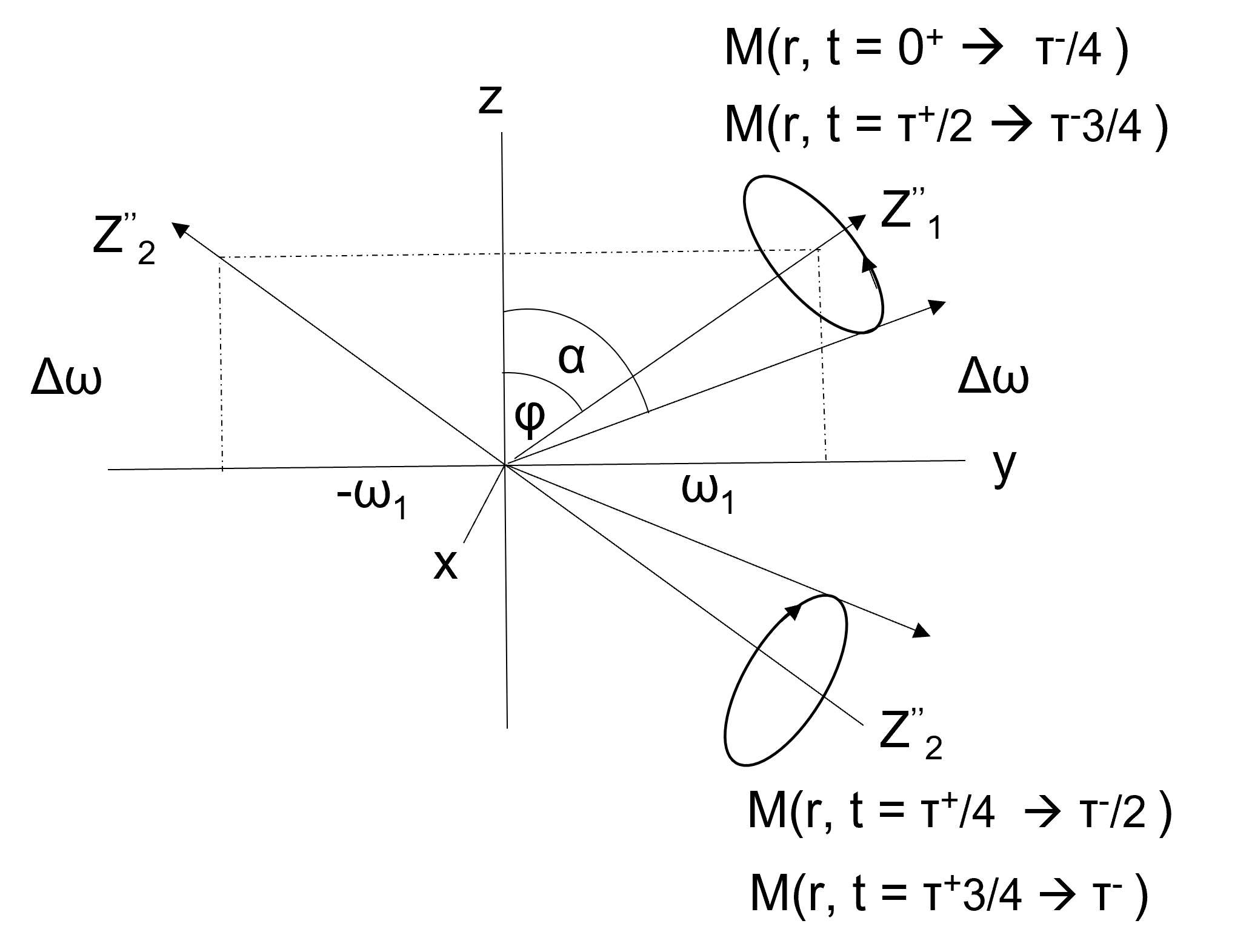
**

**Supplementary Figure S1:** Triple refocus magnetization path under on-resonance conditions. The magnetization (M(0-)) is flipped from the longitudinal axis (z-axis) with an angle α, which does not need to be 90°, towards the transverse plane (y-axis) (M(0+)) and nutates about the z_1_’’ axis (M(τ-/4)) and at time τ/4 is flipped by 2α about y-axis (M(τ+/4)) and nutates (M(τ-/2)) around z_2_’’, this trend of rotation and nutation is the same for the rest of the pulses in the train. The final magnetization (M(τ-), after full spin-locking) is brought to the negative longitudinal axis (M(τ+)) by another flip angle α. Artifacts due to imperfect 180° are compensated to an extent by the multiple refocuses.


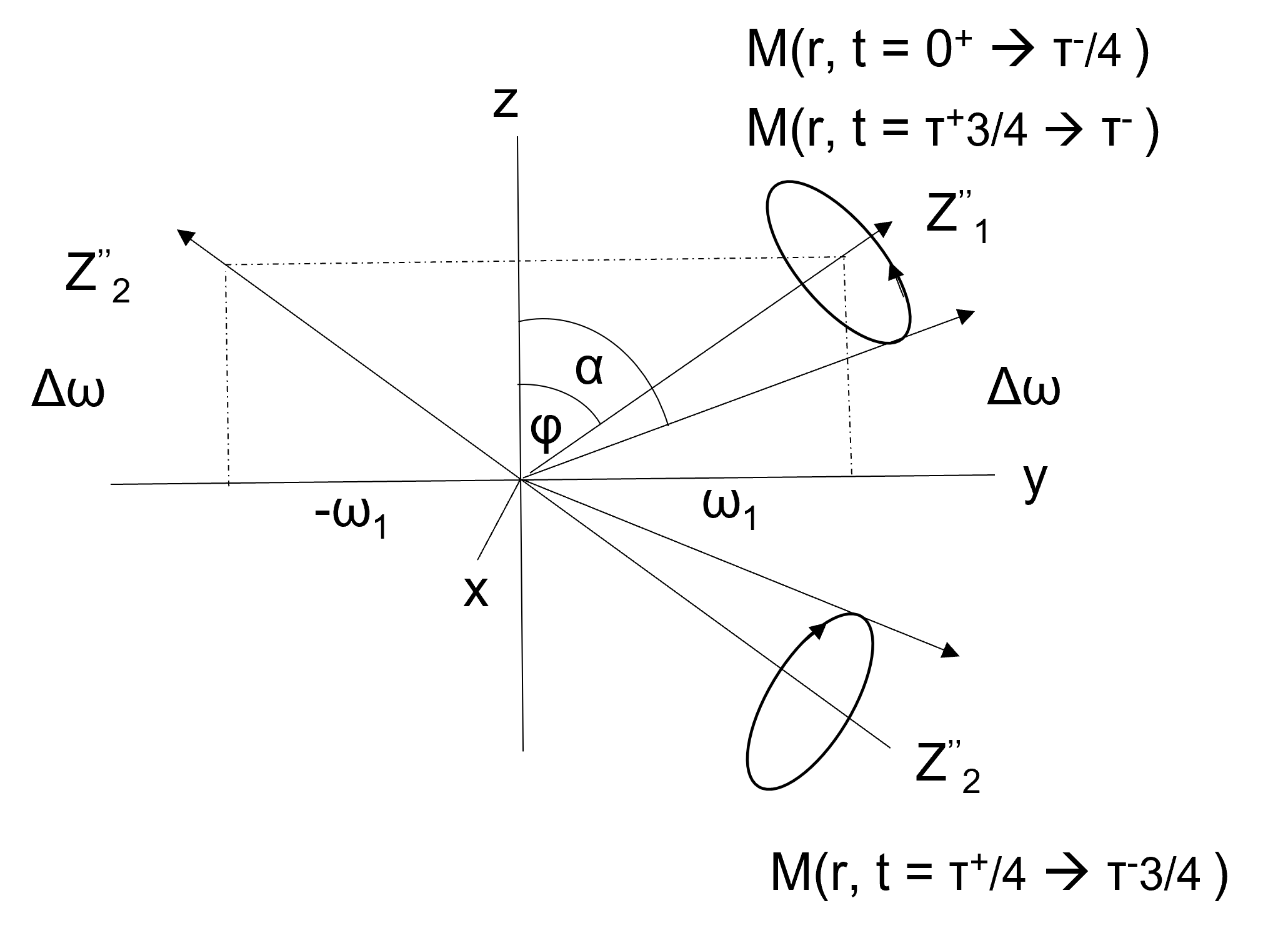


**Supplementary Figure S2:** Field insensitive spin lock with double refocusing for T1ρ relaxation measurements. Magnetization path under on-resonance conditions. The magnetization (M(0-)) is flipped from the longitudinal plane (z-axis) with an angle α, which does not need to be 90° towards the transverse plane (y-axis) (M(0+)) and nutates about the z1’’ axis (M(τ-/4)) and at time τ+/4 is flipped by 2α about y-axis (M(τ+/4)) and nutates (M(τ-3/4)) around z2’’, this trend of rotation and nutation is same for the rest of the pulses in the train. The final magnetization (M (τ-), after full spin-locking) returns to the positive longitudinal axis (M (τ+)) with another flip angle -α. Artifacts due to imperfect 180° are compensated to an extent by the double refocusing.


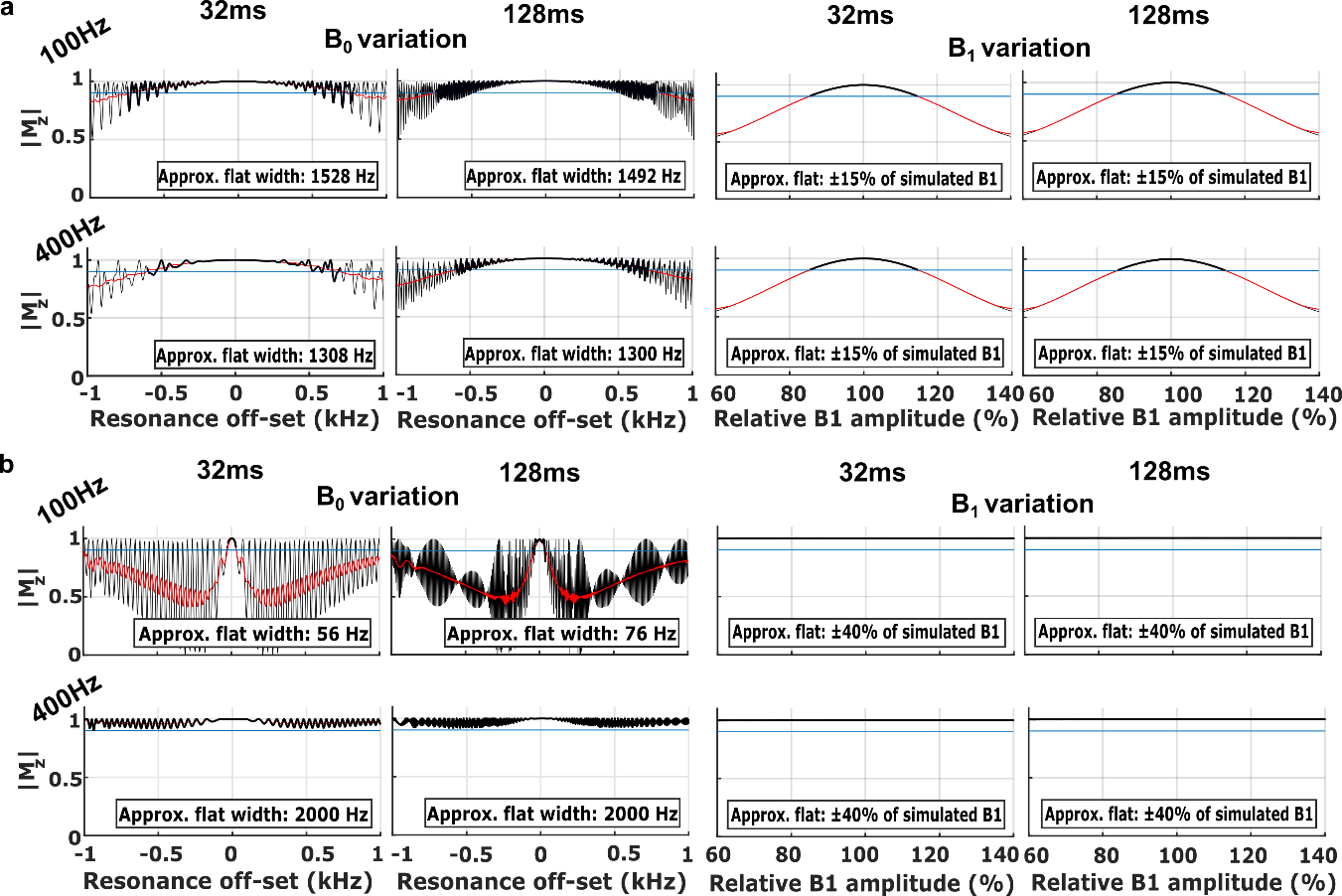


**Supplementary Figure S3:** Simulations for *PSC-SL* (a) proposed by Mitrea et al^1^, and conventional adiabatic sequence with reduced maximum power of the AHP pulse to 600 Hz (b) with the same ΔB_0_ and B_1_ field inhomogeneity ranges of up to ±1 kHz and ±40% as presented in this manuscript, for spin-lock durations of 32ms and 128 ms, and for spin-lock amplitudes of 100 Hz and 400 Hz.

**
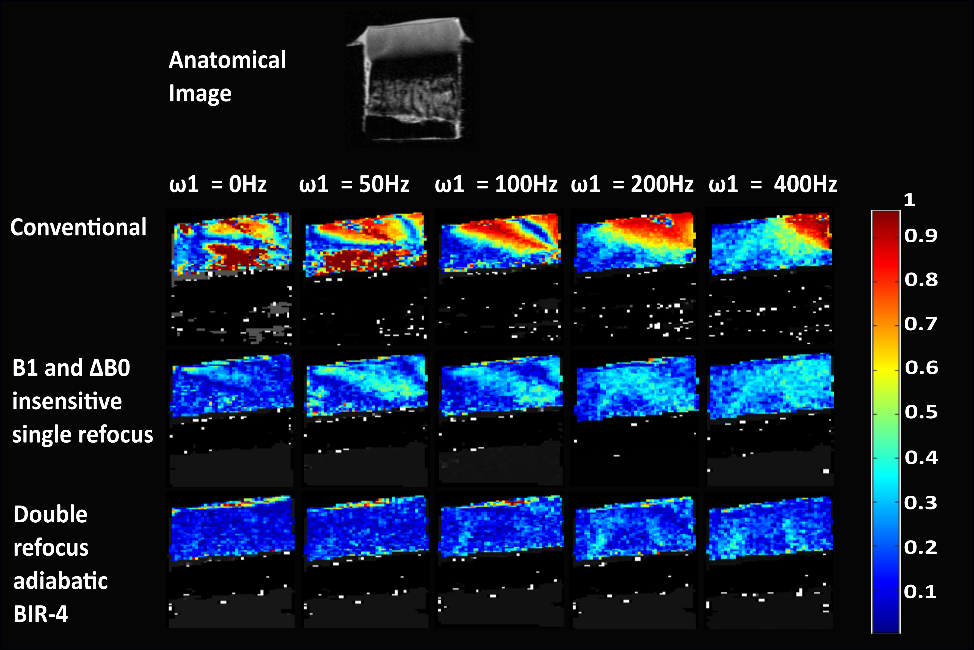
**

**Supplementary Figure S4**. Relative differences between the T1ρ maps acquired under the ideal and non-ideal conditions for the worst performing conventional (Fig. 1A), and for the two best performing, single-refocus hard pulse (Fig. 1B) and double-refocus adiabatic BIR-4 (Fig. 2C) preparation schemes at spin-lock amplitudes ranging from 0 to 400 Hz.


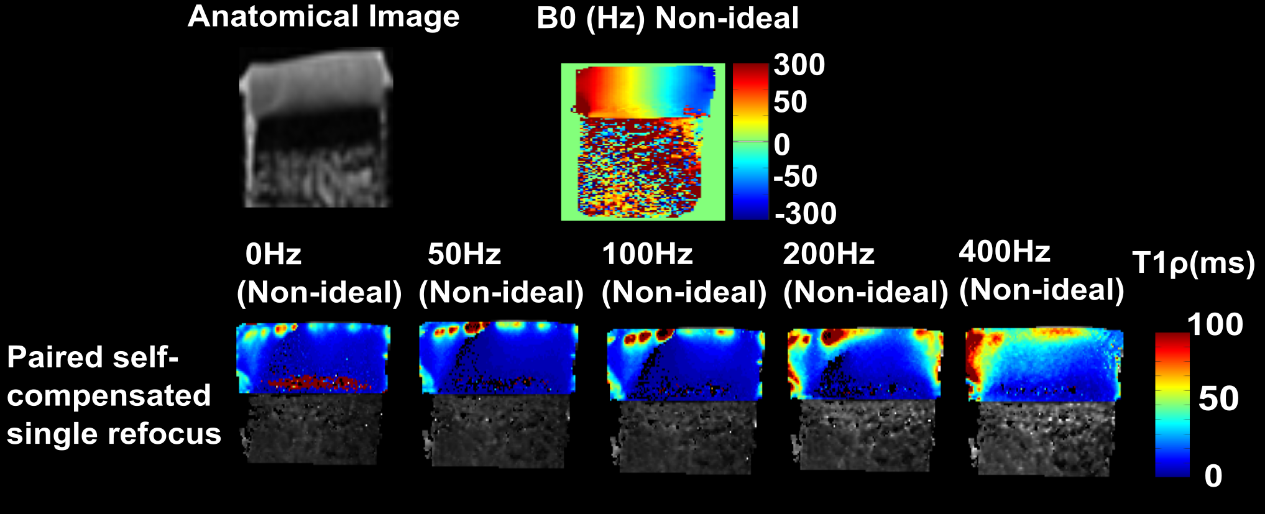


**Supplementary Figure S5**. T1ρ relaxation time maps for paired self-compensated single refocus PSC-SL sequence proposed by Mitrea et al^1^, under the non-ideal conditions for spin-lock amplitudes of 0 - 400 Hz. Anatomical reference and the corresponding B_0_ map are shown at the top.

**
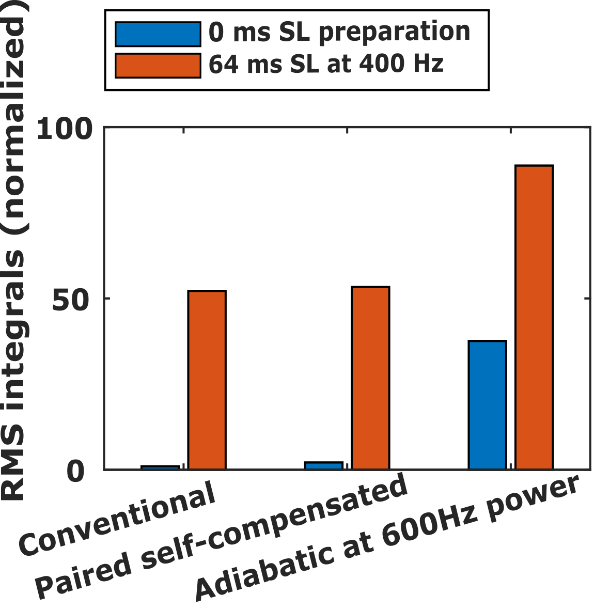
**

**Supplementary Figure S6**. Comparison of the RMS integrals (RF power) of the zero spin-lock duration RF pulse trains (blue bars) and 64 ms spin-lock duration RF pulse trains at 400 Hz spin-lock amplitude (orange bars) of the conventional pulse, paired self-compensated PSC-SL pulse sequence proposed by Mitrea et al^1^, and adiabatic CW pulse with 600 Hz maximum AHP power T_1ρ_ preparation methods, normalized to the RMS integral of the zero spin-lock duration conventional spin-lock method.


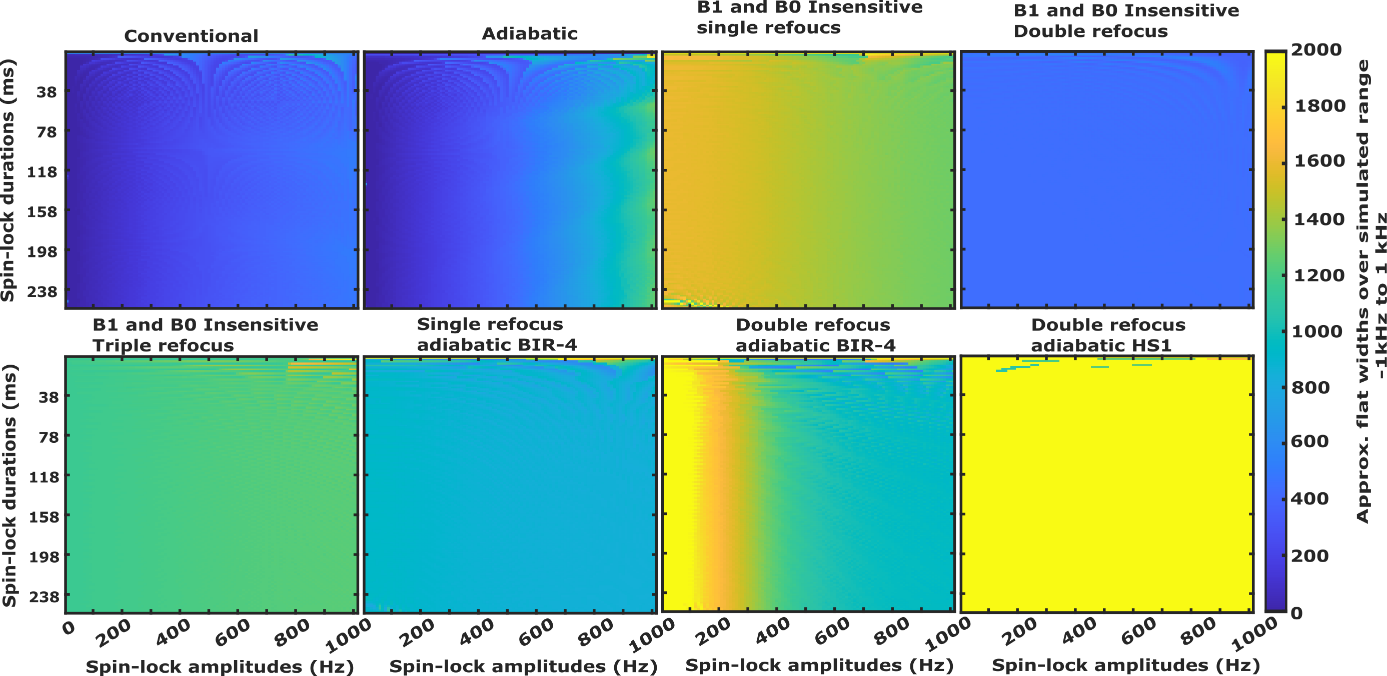


**Supplementary Figure S7.** Calculated approximate flat widths for the Bloch simulations of all the sequences in Figs. 1 and 2 over ΔB_0_ (±1 kHz) at the correct B_1_ amplitude. Simulations were run with spin-lock durations ranging from τ = 0 to 256 ms in 2 ms steps, and with spin-lock amplitudes from 0 to 1000 Hz in 10 Hz steps.


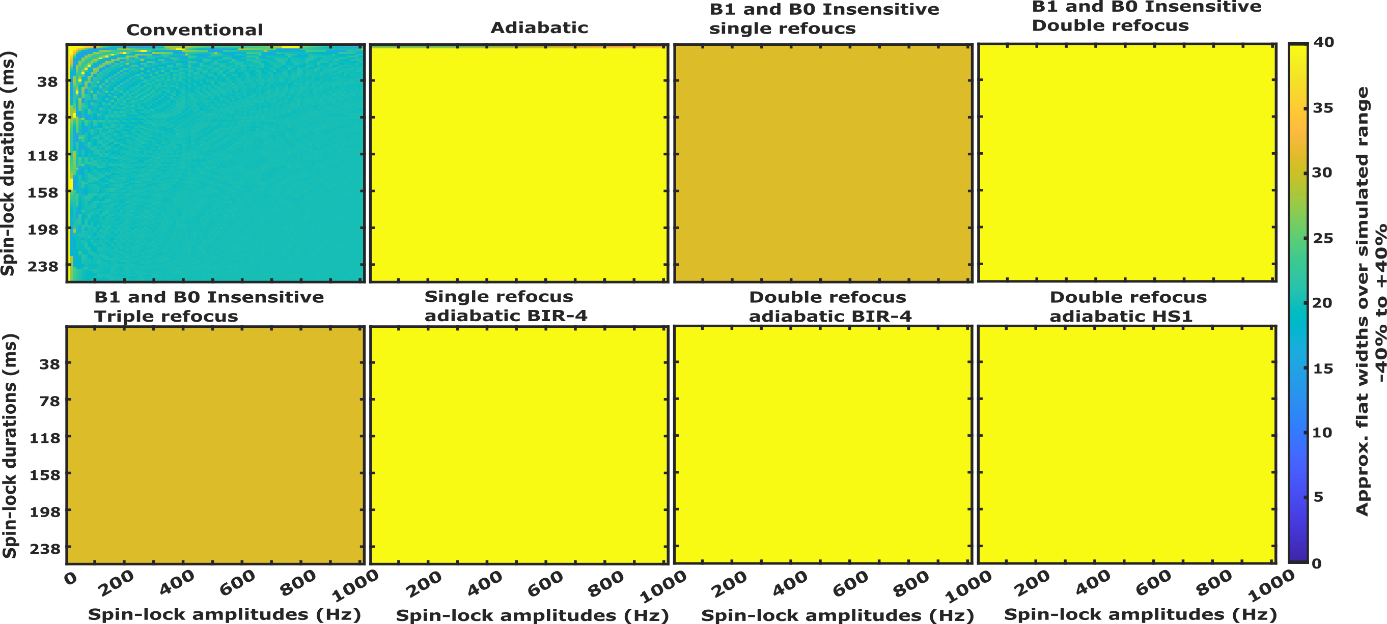


**Supplementary Figure S8.** Calculated approximate flat widths for the Bloch simulations of all the sequences in Figs. 1 and 2 over ΔB_1_ (±40%) at the correct B_0_ amplitude. Simulations were run with spin-lock durations ranging from τ = 0 to 256 ms in 2 ms steps, and with spin-lock amplitudes from 0 to 1000 Hz in 10 Hz steps.


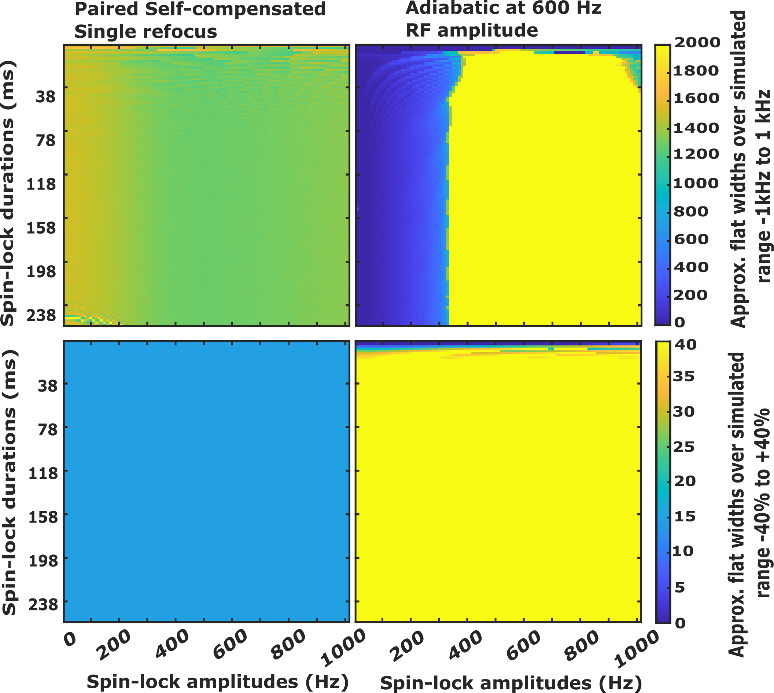


**Supplementary Figure S9:** Calculated approximate flat widths for the Bloch simulations of the *PSC-SL* proposed by Mitrea et al^1^, and for the adiabatic CW pulse with 600 Hz maximum AHP power T_1ρ_ preparation methods with similar ΔB_0_ and B_1_ field inhomogeneities of up to ±1 kHz and ±40% as presented in this manuscript. Simulations were run with spin-lock durations ranging from τ = 0 to 256 ms in 2 ms steps, and with spin-lock amplitudes from 0 to 1000 Hz in 10 Hz steps.

**Supplementary tables**

|  | Zero SL | | |  | 64 ms SL, 400 Hz |
| --- | --- | --- | --- | --- | --- |
| Preparation scheme | Pulse  length | Normalized RMS integral | RMS Amplitude |  | Normalized RMS integral |
| Conventional | 400 μs | 1 | 1250 |  | 52.2 |
| Adiabatic-CW | 8000 μs | 37.95 | 2372.1 |  | 88.8 |
| Single refocus hard pulse | 600 μs | 2.12 | 1767.8 |  | 53.3 |
| Double refocus hard pulse | 800 μs | 3.16 | 1976.4 |  | 54.3 |
| Triple refocus hard pulse | 1000 μs | 4.18 | 2091.7 |  | 55.4 |
| Single refocus adiabatic BIR-4 | 13171 μs | 61.77 | 2370.2 |  | 113 |
| Double refocus adiabatic BIR-4 | 18343 μs | 85.96 | 2369.4 |  | 137.2 |
| Double refocus adiabatic HS1 | 14060 μs | 53.61 | 1925.7 |  | 104.8 |
| Paired self-compensated pulse | 600 μs | 2.12 | 1767.8 |  | 53.32 |
| Adiabatic-CW at 600 Hz max. power | 33330 μs | 37.95 | 569.5 |  | 88.8 |
| Reference SL with 50ms, 400 Hz | 0 μs | 40 | 400 |  | - |

**Supplementary Table S1:** Pulse length, RMS integral and RMS amplitude values of preparation schemes with the zero spin-lock (SL) pulses and with 64 ms SL at 400 Hz amplitude pulses.

**References**

1. Mitrea BG, Krafft AJ, Song R, Loeffler RB, Hillenbrand CM. Paired self-compensated spin-lock preparation for improved T1ρ quantification. *J Magn Reson*. 2016;268:49-57. doi:10.1016/j.jmr.2016.04.017

2. Gram M, Seethaler M, Gensler D, Oberberger J, Jakob PM, Nordbeck P. Balanced spin-lock preparation for B1-insensitive and B0-insensitive quantification of the rotating frame relaxation time T1ρ. *Magn Reson Med*. 2021;85(5):2771-2780. doi:10.1002/mrm.28585

3. Witschey WRT, Borthakur A, Elliott MA, et al. Artifacts in T1ρ-weighted imaging: Compensation for B1 and B0 field imperfections. *J Magn Reson*. 2007;186(1):75-85. doi:10.1016/j.jmr.2007.01.015

4. Zeng, H; Daniel, G; Gatenby, C; Zhao, Y; Avison, M; Gore J. A Composite Spin-Lock Pulse For ∆ B 0 + B 1 Insensitive T 1. *Proc Int Soc Magn Reson Med*. 2006;14:2356.
